# Splicing Outcomes of 5′ Splice Site GT>GC Variants That Generate Wild-Type Transcripts Differ Significantly Between Full-Length and Minigene Splicing Assays

**DOI:** 10.3389/fgene.2021.701652

**Published:** 2021-08-05

**Authors:** Jin-Huan Lin, Hao Wu, Wen-Bin Zou, Emmanuelle Masson, Yann Fichou, Gerald Le Gac, David N. Cooper, Claude Férec, Zhuan Liao, Jian-Min Chen

**Affiliations:** ^1^Department of Gastroenterology, Changhai Hospital, Second Military Medical University, Shanghai, China; ^2^Shanghai Institute of Pancreatic Diseases, Shanghai, China; ^3^Univ Brest, Inserm, EFS, UMR 1078, GGB, Brest, France; ^4^Service de Génétique Médicale et de Biologie de la Reproduction, CHRU Brest, Brest, France; ^5^Laboratory of Excellence GR-Ex, Paris, France; ^6^Institute of Medical Genetics, School of Medicine, Cardiff University, Cardiff, United Kingdom

**Keywords:** aberrant transcript, full-length gene splicing assay, genetic variant, minigene splicing assay, splice site, SpliceAI

## Abstract

Combining data derived from a meta-analysis of human disease-associated 5′ splice site GT>GC (i.e., +2T>C) variants and a cell culture-based full-length gene splicing assay (FLGSA) of forward engineered +2T>C substitutions, we recently estimated that ∼15–18% of +2T>C variants can generate up to 84% wild-type transcripts relative to their wild-type counterparts. Herein, we analyzed the splicing outcomes of 20 +2T>C variants that generate some wild-type transcripts in two minigene assays. We found a high discordance rate in terms of the generation of wild-type transcripts, not only between FLGSA and the minigene assays but also between the different minigene assays. In the pET01 context, all 20 wild-type minigene constructs generated the expected wild-type transcripts; of the 20 corresponding variant minigene constructs, 14 (70%) generated wild-type transcripts. In the pSPL3 context, only 18 of the 20 wild-type minigene constructs generated the expected wild-type transcripts whereas 8 of the 18 (44%) corresponding variant minigene constructs generated wild-type transcripts. Thus, in the context of a particular type of variant, we raise awareness of the limitations of minigene splicing assays and emphasize the importance of sequence context in regulating splicing. Whether or not our findings apply to other types of splice-altering variant remains to be investigated.

## Introduction

In principle, both coding and intronic variants within a gene have the potential to affect splicing (Cooper et al., 2009; Scotti and Swanson, 2016; Anna and Monika, 2018; Truty et al., 2021). Genetic variants occurring within the 5′ splice site GT dinucleotide, whenever found in disease-causing or disease-predisposing genes, have generally been classified as pathogenic (Mount et al., 2019; Stenson et al., 2020). However, a problem is posed by 5′ splice site GT>GC variants (henceforth simply termed +2T>C variants) due to the fact that in the human genome, a small but nevertheless significant minority (∼1%) of introns contain the 5′ splice site GC dinucleotide (Burset et al., 2000, 2001; Abril et al., 2005; Sheth et al., 2006; Parada et al., 2014). Recently, combining data derived from a meta-analysis of human inherited disease-associated +2T>C variants and a cell culture-based Full-Length Gene Splicing Assay (FLGSA) of forward engineered +2T>C substitutions, we estimated that ∼15–18% of +2T>C variants can generate up to 84% wild-type transcripts relative to their wild-type counterparts (Lin et al., 2019). This finding was corroborated by a re-analysis (Chen et al., 2020) of the saturation genome editing data on 12 *BRCA1* +2T>C substitutions (Findlay et al., 2018).

Our aforementioned findings have two direct clinical implications. Firstly, many +2T>C variants in human disease genes that have been capable of generating some wild-type transcripts are likely to have gone largely unreported; this represents a significant deficiency in terms of our understanding of genotype-phenotype relationships and tailored treatment options given that even the minor retention of wild-type transcripts derived from a variant allele might significantly impact disease expression and severity (Ramalho et al., 2002; Den Uijl et al., 2011; Raraigh et al., 2018; Lin et al., 2019; Scalet et al., 2019; Joynt et al., 2020). In this regard, it is pertinent to mention that *CFTR* c.3873+2T>C and c.4242+2T>C transitions (Joynt et al., 2020) and *SRP68* c.184+2T>C (Schmaltz-Panneau et al., 2021) are among the most recently reported examples of disease-causing +2T>C variants that generated some wild-type transcripts. Secondly, +2T>C variants in human disease genes may not invariably be pathogenic, a notion that has received support from at least two recent publications, which reclassified *BRCA2* c.8331+2T>C (Nix et al., 2021) and *BAP1* c.783+2T>C (Goldberg et al., 2021) as variants of unknown significance.

Another important finding arising from our study was that none of the widely used splicing prediction tools were capable of reliably distinguishing those +2T>C variants that generated wild-type transcripts from those that did not (Lin et al., 2019). The root of this problem is twofold: apart from the use of GC instead of GT as the 5′ splice site dinucleotide in ∼1% of introns, these prediction tools only take into consideration short local DNA sequence motifs (Chen et al., 2020). The recently developed deep learning-based tool, SpliceAI (Jaganathan et al., 2019), performed somewhat better in this regard but was still far from perfect (Chen et al., 2020). These observations underscored the importance of experimentally determining the splicing outcomes of +2T>C variants in a clinical as well as a basic research setting. Whilst RNA analysis, using pathophysiologically relevant tissues, provides the most accurate and reliable mRNA phenotyping information on human splicing variants, this is often not possible if appropriate tissue samples are not available (Aicher et al., 2020). RNA analysis using either patient blood cells or immortalized lymphoblastoid cells represents an alternative option, providing that the gene of interest is normally expressed in these cells (Wai et al., 2020). In case of the non-feasibility of both approaches, a cell culture-based minigene splicing assay has often been devised (for some most recent examples, see Damasio et al., 2021; Hao et al., 2021; Kim et al., 2021; Kortum et al., 2021; Le Tertre et al., 2021; Morbidoni et al., 2021; Qian et al., 2021; Saint-Martin et al., 2021; Torrado et al., 2021).

Our FLGSA assay (focused on genes whose genomic sizes were < 8 kb) (Lin et al., 2019, 2020) cannot be readily used for large genes for various practical and/or technical reasons. Genome editing (Findlay et al., 2018) is a promising trend but its wide application is still some way from becoming reality. Thus, the minigene splicing assay will for the time being remain the mainstream approach for functionally characterizing potential splice-altering variants. However, an inherent drawback of the minigene splicing assay is the lack of the wider genomic sequence context of the gene under study (Zou et al., 2016; Lin et al., 2019, 2020; Tang et al., 2019). This could lead to inaccurate results and incorrect conclusions being drawn owing to the complexity of the splicing code (Fu and Ares, 2014; Drexler et al., 2020), as exemplified by the contrasting findings from the study of the *SPINK1* c.194G>A variant in a minigene assay (Beer and Sahin-Toth, 2014) and our own FLGSA assay (Wu et al., 2017). Herein, we explored whether the splicing outcomes of 20 +2T>C variants that have been previously shown to generate some wild-type transcripts by means of FLGSA and/or patient RNA analysis (Lin et al., 2019) could be replicated in two minigene assays.

## Materials and Methods

### +2T>C Variants Included for Minigene Splicing Assay and Variant Nomenclature

A total of 26 +2T>C variants were previously shown to generate some wild-type transcripts by means of FLGSA and/or patient RNA analyses (Lin et al., 2019). Of these, six variants that occurred within the first intron of their respective genes could not be readily analyzed by the minigene assay and hence were excluded from further consideration.

All the remaining 20 variants were included in the current analysis (Table 1). Of these, six had been originally reported to be both naturally occurring and disease causing. These six pathogenic variants included the five variants that had previously been demonstrated to generate some wild-type transcripts by means of patient RNA analysis (i.e., *CD3E* IVS7+2T>C, *CD40LG* IVS3+2T>C, *DMD* IVS54+2T>C, *PLP1* IVS5+2T>C and *SPINK1* IVS3+2T>C) plus *HBB* IVS2+2T>C. Although the latter *HBB* IVS2+2T>C variant had no accompanying patient RNA data, it was suggested to have had a limited impact on splicing due to its associated hematological phenotype that was milder than would have been expected from a null allele (Frischknecht et al., 2009); its orthologous counterpart in the rabbit *Hbb* gene has been experimentally shown to generate wild-type transcripts (Aebi et al., 1986, 1987); and the human variant was also shown to generate wild-type transcripts in a FLGSA assay (Lin et al., 2019). Of the five pathogenic variants subjected to patient RNA analysis, only *SPINK1* IVS3+2T>C was also analyzed by FLGSA; the findings from the patient RNA analysis (Kume et al., 2006) and FLGSA assay (Zou et al., 2016; Lin et al., 2019) were remarkably similar.

**TABLE 1:** Results from minigene splicing analyses of 20 +2T>C variants that were previously reported to generate wild-type transcripts.

**Gene**	**mRNA reference**	**Variant^a^**	**Summary of previous data**		**Current results^c^**	
			**Analytical method employed**	**Expression level of the wild-type transcript^b^**	**pET01**	**pSPL3**
*CD3E*	NM_000733.3	IVS7+2T>C	Patient RNA analysis	1-5%	–	–
*CD40LG*	NM_000074.2	IVS3+2T>C	Patient RNA analysis	15%	+	+
*DBI*	NM_001079862.2	IVS2+2T>C	FLGSA^d^	Not quantified	+	–
*DMD*	NM_004006.2	IVS54+2T>C	Patient RNA analysis	10%	+	+
*DNAJC19*	NM_145261.3	IVS5+2T>C	FLGSA	42%	+	×
*FOLR3*	NM_000804.3	IVS4+2T>C	FLGSA	Not quantified	+	+
*HBB*	NM_000518.5	IVS2+2T>C	FLGSA	Not quantified	+	–
*IFNL2*	NM_172138.1	IVS5+2T>C	FLGSA	5%	+	+
*IL10*	NM_000572.3	IVS3+2T>C	FLGSA	Not quantified	+	+
*MGP*	NM_000900.4	IVS2+2T>C	FLGSA	80%	–	–
*PLP1*	NM_000533.4	IVS5+2T>C	Patient RNA analysis	8%	–	–
*PSMC5*	NM_001199163.1	IVS6+2T>C	FLGSA	56%	–	–
		IVS8+2T>C	FLGSA	56%	–	–
		IVS10+2T>C	FLGSA	46%	+	–
*RPL11*	NM_000975.5	IVS2+2T>C	FLGSA	Not quantified	+	+
		IVS3+2T>C	FLGSA	Not quantified	+	+
*RPS27*	NM_001030.4	IVS2+2T>C	FLGSA	63%	–	–
		IVS3+2T>C	FLGSA	Not quantified	+	×
*SELENOS*	NM_203472.2	IVS5+2T>C	FLGSA	14%	+	+
*SPINK1*	NM_003122.3	IVS3+2T>C	Patient RNA analysis/FLGSA	10%	+	–

*^a^In accordance with the traditional IVS (intervening sequence or intron) nomenclature as previously described (Lin et al., 2019, 2020). See Supplementary Table 1 for additional information including specifically the HGVS nomenclature (den Dunnen et al., 2016).*

*^b^Expression level of the wild-type transcripts generated from the variant allele relative to that (set as 1) generated from the wild-type allele (see Lin et al., 2019; Chen et al., 2020).*

*^c^+, the variant generated wild-type transcripts; –, the variant did not generate wild-type transcripts; ×, the variant was not analyzed by the minigene assay owing to the non-expression of the wild-type transcripts from the corresponding wild-type minigene vectors.*

*^d^FLGSA, full-length gene splicing assay.*

The remaining 14 variants included in this study were not known to be disease causing at the time (Lin et al., 2019). They represent forward engineered +2T>C substitutions, all being found to generate wild-type transcripts by means of FLGSA (Lin et al., 2019).

For ease of description and to be consistent with our previous publications (Lin et al., 2019, 2020; Chen et al., 2020), all included +2T>C variants were described in accordance with the traditional IVS (intervening sequence or intron) nomenclature (Table 1). Their respective chromosome locations, hg38 coordinates, reference alleles in hg38 and HGVS nomenclature (den Dunnen et al., 2016) are, however, provided in Supplementary Table 1.

### Construction of pET01 and pSPL3 Wild-Type Minigene Expression Vectors by Means of In-Fusion Cloning

**FIGURE 1:**
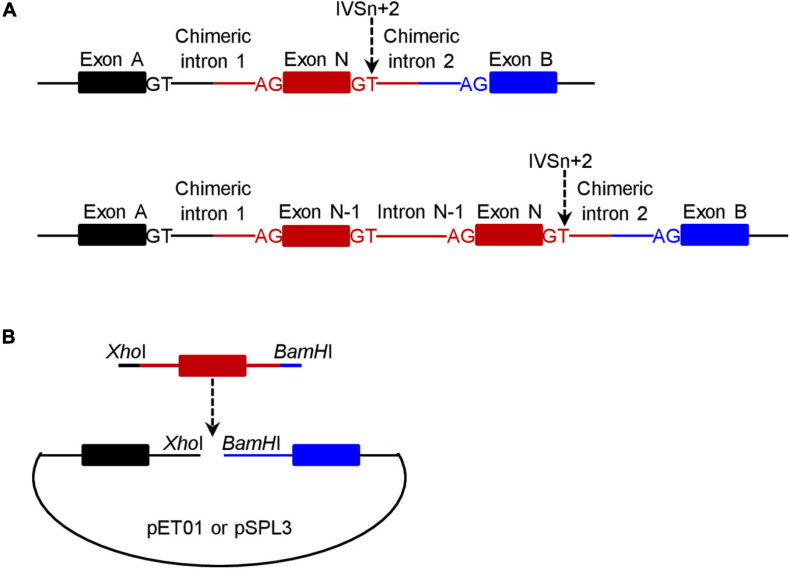
Schematic illustrations of the minigene expression constructs in the context of wild-type inserts. **(A)** Illustration of the target gene sequence (in red) inserted into the pET01 and pSPL3 exon trapping vectors. The number of the +2T>C variant-affected intron is N and the IVSn+2T site is indicated by a downward pointing arrow. For most variants (*n* = 18), the insert comprised a single exon (i.e., exon N) plus flanking intronic sequences on both sides (upper panel). For two variants, the insert comprised two exons (exon N-1 and exon N) plus flanking and intervening intronic sequences (lower panel). The two exons (exon A and exon B) located within the vector and the canonical splice donor GT and acceptor AG sites defining the two chimeric introns (upper panel) or the two chimeric introns and intron N-1 (lower panel) are also denoted in the figure. **(B)** Illustration of how a wild-type minigene expression vector was constructed. The insert (in red) was PCR amplified with 5′ *Xho*I-harboring and 3′ *BamH*I-harboring in-fusion primers with respect to the pET01 or pSPL3 vector. The resulting PCR products were inserted into their respective linearized vectors by means of in-fusion cloning.

For a given +2T>C variant, the corresponding wild-type genomic sequences cloned into the pET01 and pSPL3 exon trapping vectors were always identical. Of the 20 wild-type inserts, 18 comprised 63-330 bp sequence from the 3′ end of N-1 intron, the entire exon N and 65–328 bp sequence from the 5′ end of intron N (N is the number of the variant-affected intron) (see upper panel in Figure 1A). The other two wild-type inserts (for *PSMC5* IVS10+2T>C and *SELENOS* IVS5+2>C, respectively) instead comprised 50–116 bp sequence from intron N-2, entire exon N-1, entire intron N-1, entire exon N and 96–294 bp sequence from intron N (lower panel in Figure 1A); this was done primarily due to the small size (<100 bp) of the respective intron N-1 in these two cases. See Supplementary Table 2 for the sequences of all inserts.

Two pairs of 5′ *Xho*I-harboring and 3′ *BamH*I-harboring primers, one for in-fusion cloning into the pET01 trapping vector and the other for in-fusion cloning into the pSPL3 exon trapping vector, were designed to amplify each insert (Figure 1B). Primer sequences are provided in Supplementary Table 3. PCR was performed in a 25 μL reaction mixture containing 0.5 U KAPA HiFi HotStart DNA Polymerase (Kapa Biosystems), 0.75 μL KAPA dNTP Mix (300 μM final), 5 μL 5× KAPA HiFi Buffer, 50 ng DNA (from a healthy Chinese subject), and 0.3 μM forward and reverse primers. The PCR program comprised an initial denaturation at 95°C for 5 min, followed by 30 cycles of denaturation at 98°C for 20 s, annealing at 66°C for 15 s, extension at 72°C for 1 min, and a final extension at 72°C for 5 min.

PCR products of the expected size were purified with the Cloning Enhancer kit (TaKaRa). The purified products were then cloned into the *Xho*I and *BamH*I restriction sites of the linearized pET01 or pSPL3 vector with the In-Fusion HD Cloning kit (TaKaRa) according to the manufacturer’s instructions. Transformation was performed using Stellar Competent Cells (TaKaRa). Transformed cells were spread onto LB agar plates with 50 μg/mL ampicillin and incubated at 37°C overnight. Plasmid constructs containing inserts were confirmed by Sanger sequencing.

Primers were designed by our laboratory in the Changhai Hospital. Primer synthesis, insert amplification, in-fusion cloning and verification of the inserted fragments were all performed by GENEWIZ, Beijing, China.

### Generation of pET01 and pSPL3 +2T>C Variant Minigene Expression Vectors by Means of Site-Directed Mutagenesis

+2T>C variants were introduced into their respective wild-type minigene expression constructs by means of the QuikChange II XL Site-Directed Mutagenesis Kit (Agilent Technologies). Mutagenesis, transformation, plasmid preparation and validation of the introduced variants were performed as previously described (Lin et al., 2019). Sequences of the mutagenesis primers are provided in Supplementary Table 4.

### Cell Culture, Transfection, RNA Extraction, and Reverse Transcription

These were performed as previously described (Lin et al., 2019).

### Reverse Transcription-Polymerase Chain Reaction (RT-PCR) Analysis

RT-PCR was performed in a 25-μL reaction mixture containing 12.5 μL HotStarTaq Master Mix (Qiagen), 1 μL cDNA, and 0.4 μM each primer (5′-GAGGGATCCGCTTCCTGGCCC-3′ (forward) and 5′-CTCCCGGGCCACCTCCAGTGCC-3′ (reverse) for pET01 expression vectors (both primers are located within the pET01 vector sequence); 5′-TCTGAGTCACCTGGACAACC-3′ (forward) and 5′-ATCTCAGTGGTATTTGTGAGC-3′ (reverse) for pSPL3 expression vectors (both primers are located within the pSPL3 vector sequence)). The PCR program had an initial denaturation step at 95°C for 15 min, followed by 30 cycles of denaturation at 94°C for 45 s, annealing at 58°C for 45 s, extension at 72°C for 1 min/kb, and a final extension step at 72°C for 10 min. RT-PCR products of a single band were cleaned by ExoSAP-IT (Affymetrix). In the case of multiple bands, the bands were excised from the agarose gel and then purified by QIAquick Gel Extraction Kit (Qiagen). Sequencing primers were those used for the RT-PCR analyses and sequencing was performed using the BigDye Terminator v1.1 Cycle Sequencing Kit (Applied Biosystems).

## Results

### Rationale of Experimental Protocol

**FIGURE 2:**
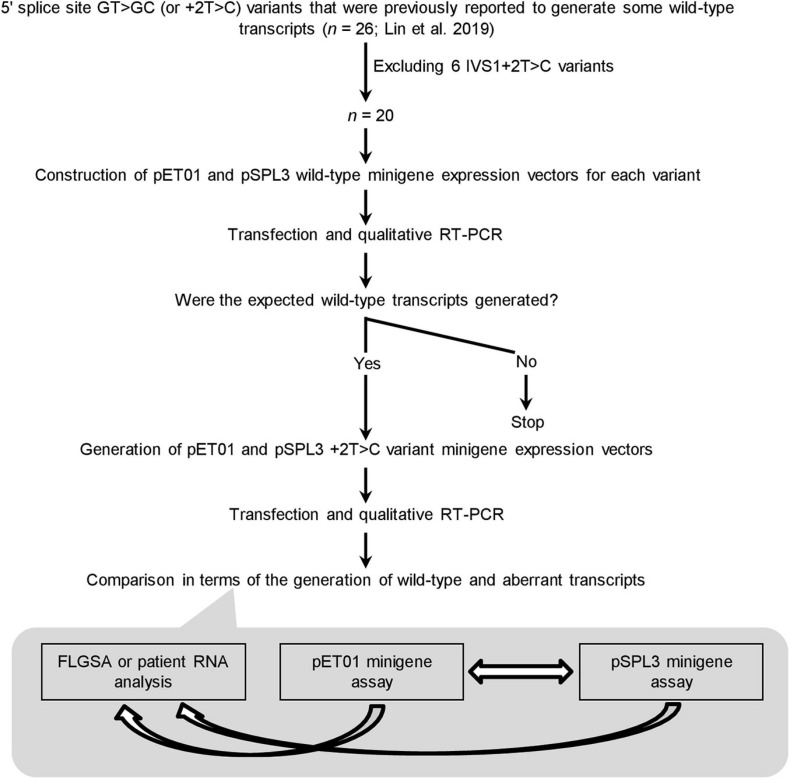
Outline of the experimental procedures. IVS, intervening sequence or intron; RT-PCR, reverse transcription-polymerase chain reaction. FLGSA, full-length gene splicing assay.

The experimental procedures adopted in this study are summarized in Figure 2. Before presenting the results obtained in some detail, we would like to summarize our protocol in terms of its four components.

First, the 20 +2T>C variants included for minigene assay analysis represent the totality of the variants that were previously found to generate wild-type transcripts as assessed by FLGSA and/or patient RNA analyses excluding all IVS1+2T>C ones (Lin et al., 2019). (NB. Intronic variants located near the first or last exon of the gene cannot be readily evaluated by a minigene assay without special adaptation (Chen et al., 2018; Raud et al., 2019; Tang et al., 2019).) The accuracy and reliability of the FLGSA-obtained functional assessment of the +2T>C variants have been extensively addressed in our previous publications (Lin et al., 2019, 2020; Chen et al., 2020).

Second, for each +2T>C variant under study, the corresponding wild-type genomic sequences inserted into the two minigene vectors, pET01 and pSPL3, were always identical.

Third, the generation (or not) of wild-type transcripts—determined by qualitative RT-PCR analyses as previously described (Lin et al., 2019)—was used as the basis for comparison.

Fourth, a wild-type transcript refers to the product containing precisely Exon A, Exon N and Exon B or Exon A, Exon N-1, Exon N and Exon B as depicted in Figure 1A. The authenticity of all wild-type transcripts was confirmed by Sanger sequencing. Most aberrant transcripts were also Sanger sequenced.

### Generation (or Not) of Wild-Type Transcripts

#### Two Exceptions to the Rule That Wild-Type Minigene Constructs Invariably Express the Expected Wild-Type Transcripts

Of the 40 wild-type minigene constructs (20 in the pET01 context and 20 in the pSPL3 context), only two did not express the expected wild-type transcripts (Supplementary Figures 1–20), both of them in the pSPL3 context. Specifically, the pSPL3 *DNAJC19* IVS5+2T minigene construct expressed a transcript lacking *DNAJC19* exon 5 but containing instead a 118-bp pseudoexon (Figure 3). The pSPL3 *RPS27* IVS3+2T minigene construct expressed a transcript with *RPS27* exon 3 being skipped (Supplementary Figure 18). These two wild-type minigene constructs were thus not mutated to their corresponding variant versions. In other words, *DNAJC19* IVS5+2T>C and pSPL3 *RPS27* IVS3+2T>C were not analyzed by the minigene assay in the pSPL3 context.

**FIGURE 3:**
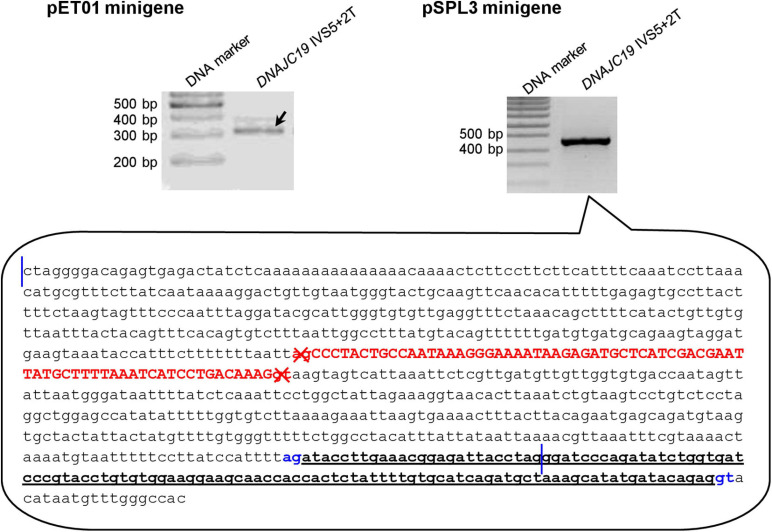
Reverse transcription-polymerase chain reaction results from minigene assays with respect to the *DNAJC19* IVS5+2T (i.e., wild-type) construct. In the pET01 minigene assay, the *DNAJC19* IVS5+2T construct yielded the expected wild-type transcripts (indicated by the left oblique downward pointing arrow). In the pSPL3 minigene assay, the *DNAJC19* IVS5+2T construct yielded an aberrant transcript, whose nature was illustrated in the call-out box. In the call-out box, the sequence delimited by the two vertical blue lines refers to the entire wild-type *DNAJC19* DNA insert, which comprised exon 5 (in upper case and in red) and partial intronic sequences on both sides. The sequence downstream of the second vertical line refers to partial downstream pSPL3 vector sequence. The aberrant transcript did not contain *DNAJC19* exon 5 but instead contained a 118-bp pseudoexon (in bold and underlined) that spanned the chimeric region of the chimeric intron 2 (see Figure 1A for term definition). The aberrantly inactivated AG-GT splice sites flanking *DNAJC19* exon 5 are highlighted in red and denoted by crosses. The aberrantly activated cryptic AG-GT splice sites are highlighted in blue. See Supplementary Figure 5 for the full gel photographs.

#### Four +2T>C Variants Exhibited Discordance Between the Two Minigene Assays

**FIGURE 4:**
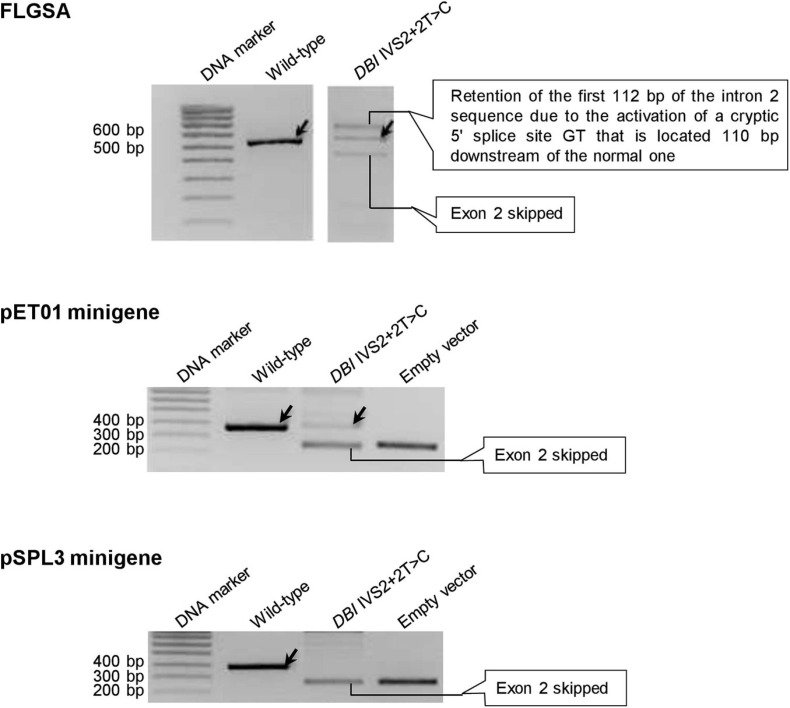
Reverse transcription-polymerase chain reaction (RT-PCR) results from the pET01 and pSPL3 minigene assays with respect to the *DBI* IVS2+2T>C variant. Results from the previously performed full-length gene splicing assay (FLGSA) (Lin et al., 2019) are included for the sake of comparison (NB. The two aberrant transcripts were newly sequenced in this study). In all panels, wild-type transcripts are indicated by oblique downward pointing arrows. See Supplementary Figure 3 for the full gel photographs with respect to the minigene assays. The FLGSA data were adapted from Lin et al. (2019) with permission (Copyright 2020 Wiley Periodicals LLC).

Four variants, *DBI* IVS2+2T>C (Figure 4), *HBB* IVS2+2>C (Supplementary Figure 7), *PSMC5* IVS10+2T>C (Supplementary Figure 14) and *SPINK1* IVS3+2T>C (Supplementary Figure 20), generated wild-type transcripts in the pET01 minigene assay but not in the pSPL3 minigene assay.

#### Six +2T>C Variants Failed to Generate Wild-Type Transcripts in Both Minigene Assays

**FIGURE 5:**
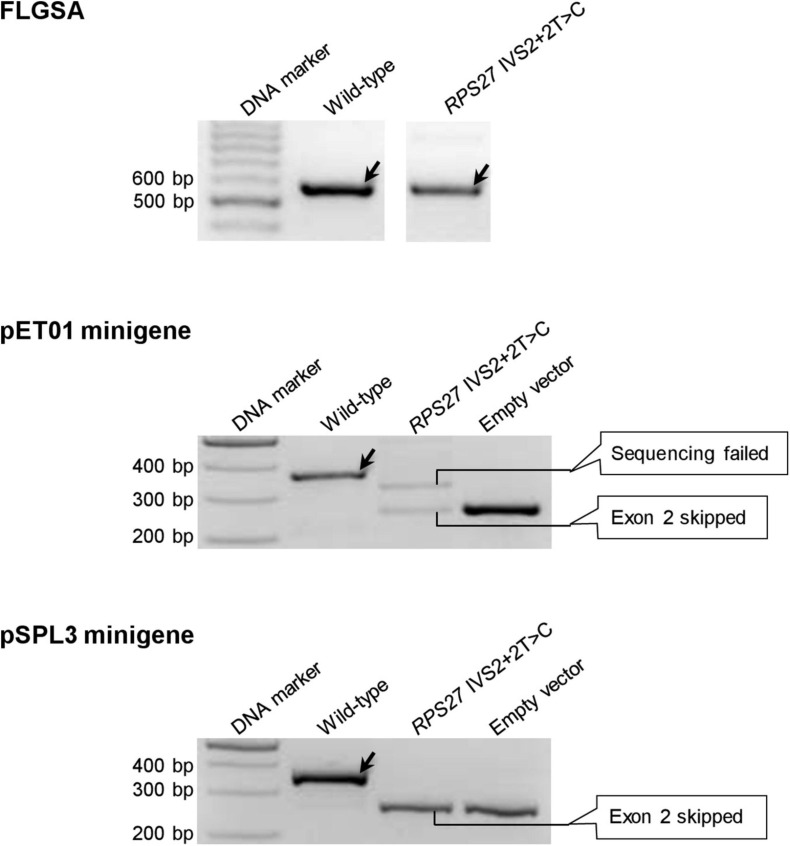
Reverse transcription-polymerase chain reaction results from the pET01 and pSPL3 minigene assays with respect to the *RPS27* IVS2+2T>C variant. Results from the previously performed full-length gene splicing assay (FLGSA) (Lin et al., 2019) are included for the sake of comparison. In all panels, wild-type transcripts are indicated by oblique downward pointing arrows. See Supplementary Figure 17 for the full gel photographs with respect to the minigene assays. The FLGSA data were adapted from Lin et al. (2019) with permission (Copyright 2020 Wiley Periodicals LLC).

The six variants are *CD3E* IVS7+2T>C (Supplementary Figure 1), *MGP* IVS2+2T>C (Supplementary Figure 10), *PLP1* IVS5+2T>C (Supplementary Figure 11), *PSMC5* IVS6+2T>C (Supplementary Figure 12), *PSMC5* IVS8+2T>C (Supplementary Figure 13) and *RPS27* IVS2+2T>C (Figure 5).

#### Eight Variants Generated Wild-Type Transcripts in Both Minigene Assays

**FIGURE 6:**
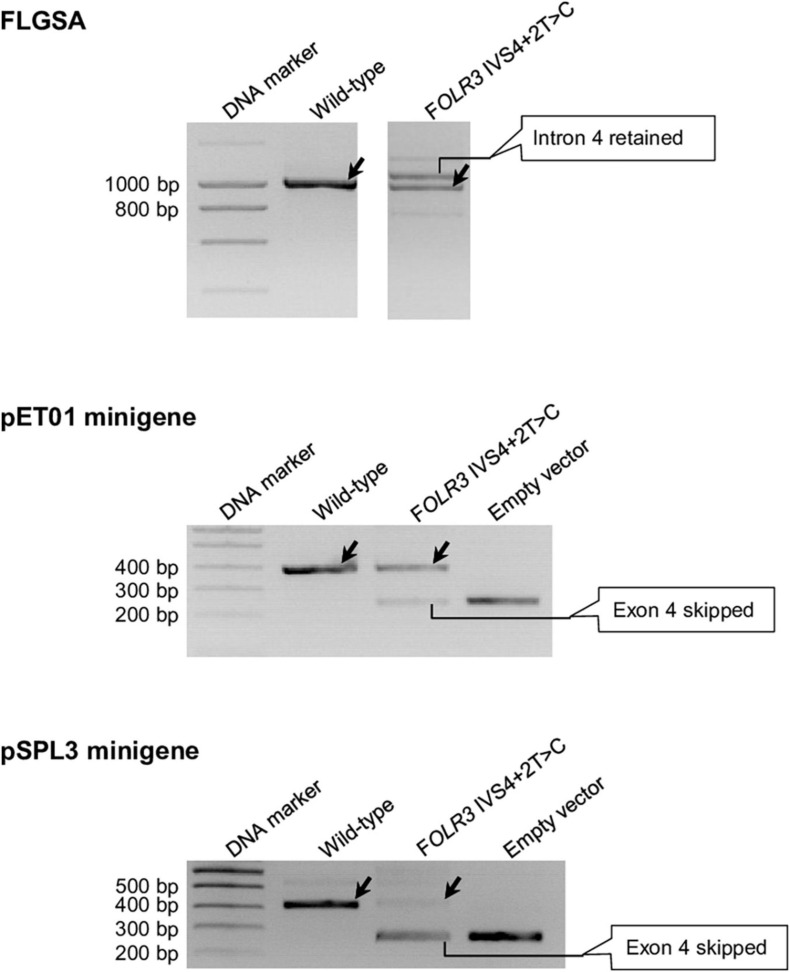
Reverse transcription-polymerase chain reaction results from the pET01 and pSPL3 minigene assays with respect to the *FOLR3* IVS4+2T>C variant. Results from the previously performed full-length gene splicing assay (FLGSA) (Lin et al., 2019) are included for the sake of comparison (NB. the aberrant transcript with retained intron 4 was newly sequenced in this study). In all panels, wild-type transcripts are indicated by oblique downward pointing arrows. See Supplementary Figure 6 for the full gel photographs with respect to the minigene assays. The FLGSA data were adapted from Lin et al. (2019) with permission (Copyright 2020 Wiley Periodicals LLC).

These eight variants are *CD40LG* IVS3+2T>C (Supplementary Figure 2), *DMD* IVS54+2T>C (Supplementary Figure 4), *FOLR3* IVS4+2T>C (Figure 6), *IFNL2* IVS5+2T>C (Supplementary Figure 8), *IL10* IVS3+2T>C (Supplementary Figure 9), *RPL11* IVS2+2T>C (Supplementary Figure 15), *RPL11* IVS3+2T>C (Supplementary Figure 16) and *SELENOS* IVS5+2T>C (Supplementary Figure 19).

#### Synthesis

The above findings are summarized in Table 1. In short, in the pET01 context, all 20 wild-type minigene constructs generated the expected wild-type transcripts; of the 20 corresponding variant minigene constructs, 14 (70%) generated wild-type transcripts. In the pSPL3 context, only 18 of the 20 wild-type minigene constructs generated the expected wild-type transcripts; of the 18 corresponding variant minigene constructs, only 8 (44%) generated wild-type transcripts.

## Discussion

In this study, we set out to systematically analyze the splicing outcomes of 20 +2T>C variants that had been previously shown to generate varying levels of wild-type transcripts by means of FLGSA and/or patient RNA analyses (Lin et al., 2019), in two minigene systems. We found a fairly high level of discordance between the different systems in terms of the generation of wild-type transcripts (Table 1). First and foremost, 30% (*n* = 6) of the 20 +2T>C variants analyzed in the pET01 minigene assay and 56% (*n* = 10) of the 18 +2T>C variants analyzed in the pSPL3 minigene assay failed to generate wild-type transcripts. It would thus appear that the minigene assays have a tendency to exaggerate the negative effect of the +2T>C variants on splicing. Whether this is bound up with the artificiality of the minigene structure or simply represents a chance finding, remains to be established. In line with our own findings, the aforementioned reclassified *BRCA2* c.8331+2T>C variant (Nix et al., 2021) had been previously found to generate no wild-type transcripts at all by means of a minigene assay (Fraile-Bethencourt et al., 2017). However, using exonic tag-SNP analysis of transcripts expressed in Epstein-Barr virus-immortalized lymphoblastoid cells from a heterozygous *BRCA2* c.8331+2T>C carrier, Gelli et al. (2019) demonstrated that wild-type transcripts were derived from both the wild-type and c.8331+2T>C alleles, although they did not specify the relative levels of wild-type transcript emanating from the wild-type and variant alleles. More recently, using exonic tag-SNP analysis of transcripts expressed in blood cells from a *BRCA2* c.8331+2T>C heterozygote, Nix et al. (2021) demonstrated that 62 and 38% of the wild-type transcripts were derived from the wild-type and variant alleles, respectively. For the purpose of comparison, *BRCA2* c.68-7T>A, which causes an ∼20% functional loss of the variant allele, has been firmly established to be nonpathogenic (Colombo et al., 2018) whilst analysis of a neutral leaky variant (c.231T>G) has served to demonstrate that a reduction of ∼60% of full-length *BRCA2* transcripts from the mutant allele does not give rise to any measurable increase in cancer risk (Tubeuf et al., 2020).

Significant discordance was also apparent between the two minigene systems, into which identical inserts were cloned for each variant under study, in terms of the results obtained (Table 1). Moreover, even in the cases that showed concordance in terms of the generation (or not) of wild-type transcripts, the splicing outcomes may have differed in terms of the nature of the aberrant transcripts and/or relative levels of the wild-type transcripts. Take, for example, the *FOLR3* IVS4+2T>C variant that generated wild-type transcripts in all three systems: the aberrant transcripts generated from FLGSA were different from those generated from the two minigene assays; moreover, the relative levels of the wild-type transcript were markedly different between the two minigene assays. Specifically, the FLGSA-derived aberrant transcript had retained intron 4 whereas the minigene-derived aberrant transcript had skipped exon 4; further, the level of the pET01-derived wild-type transcripts was much higher than that of the pSPL3-derived wild-type transcripts, as indicated by the relative intensities of the wild-type and aberrant transcript bands (Figure 6).

All the above mentioned discordant findings could be attributed primarily (if not solely) to differences in the underlying sequence contexts because our previous FLGSA and the current minigene assays were all performed under the same experimental conditions and employing the same procedures. As such, the high level of discordant findings between the different systems used should not be regarded as surprising given that (i) the sequence determinants for the 5′ splice site go beyond the best studied 9-bp consensus sequence motif (see Lin et al., 2019 and references therein) and (ii) splicing is a complicated as well as a coordinated process across different introns (Fu and Ares, 2014; Drexler et al., 2020). In this context, it is pertinent to cite a previous study, in which two splicing reporter minigenes were found to exhibit very different sensitivities in relation to the effects of 13 *MLH1* variants on exon 10 skipping; it was the one that most closely approximated the pattern of exon 10 skipping *in vivo* (in the context of the wild-type *MLH1* exon 10 minigene construct) that was used for the final analysis (Soukarieh et al., 2016). Taken together with our current findings, this indicates that it is most unlikely that a universal splicing reporter minigene could ever be developed that would be suitable for the analysis of all splicing variants. In other words, different exon trapping vectors carrying a particular wild-type target gene insert might need to be tested in advance with a view to selecting one empirically that most closely resembled the normal expression pattern of the gene designated for functional analysis. Alternatively, a midigene splicing assay (Sangermano et al., 2018) might be considered with a view to increasing the natural sequence context of the variant under study.

Although we have provided experimental evidence that genomic sequence context has influenced the splicing outcome of +2T>C variants capable of generating wild-type transcripts, it is beyond our current ability to discern precisely how and why these differences originated. For illustrative purposes, let us take the two pSPL3 wild-type minigene constructs that did not generate the expected wild-type transcripts. First, the pSPL3 *DNAJC19* IVS5+2T minigene construct expressed a transcript lacking *DNAJC19* exon 5 and containing instead a 118-bp pseudoexon. As shown in Figure 3, this was due to the inactivation of the physiological GT-AG splice sites defining *DNAJC19* exon 5 and the concurrent activation of cryptic splice sites located within the chimeric intron 2. However, based upon comparisons with the 3′ splice site consensus sequence (CAG|G) and 5′ splice site consensus sequence (MAG|GTRAGT where M is A or C and R is A or G)^1^, we could not draw any meaningful conclusions about the alternative use of the *DNAJC19* exon 5-defining GT-AG splice sites and the aberrantly activated cryptic GT-AG splice sites observed in the minigene construct. Second, the pSPL3 *RPS27* IVS3+2T minigene construct expressed an *RPS27* transcript skipping exon 3 (Supplementary Figure 18). One might argue that this could somehow be associated with alternative splicing. However, although *RPS27* has three alternative transcripts,^2^ exon 3 (in the context of NM_001030.6) is common to all three. Even if *RPS27* exon 3 was differentially used by the three transcripts, the fact of its being skipped only in the *pSPL3* context points to differences in sequence that extend beyond the gene inserts.

There is one final point to make. Although we have provided experimental evidence that points to limitations in the minigene-based analysis of splicing, our findings should not be interpreted as a challenge to the current preeminence of the minigene splicing assay which is one of the most widely used analytical tools employed for the interpretation of potentially pathogenic variants. Indeed, all methods for assessing splicing have their advantages and inconveniences.

Our study has its limitations. For example, we used only one cell line for transfection and subsequent RT-PCR analysis. It would be interesting to see whether the same results were obtained employing another cell line. Here it may nonetheless be pertinent to mention that in our previous study, we have analyzed 10 +2T>C substitutions that generated wild-type transcripts and 10 +2T>C substitutions that did not generate wild-type transcripts in HEK293T cells for FLGSA in HeLa cells; we observed entirely consistent findings in the two cell lines in terms of the generation of wild-type transcripts or not (Lin et al., 2019). Moreover, in common with our previous studies (Lin et al., 2019, 2020), our findings were based on qualitative RT-PCR/gel analysis in terms of the absence or presence of the wild-type transcripts. Repeating the experiments using another more precise method would strengthen our findings. However, we believe that since an aberrant transcript band was always generated by the variant minigene expression vectors (or in other words, the aberrant transcripts always served as an internal control for gene expression), our findings should be highly reliable.

## Conclusion

Our study provides experimental evidence that +2T>C variants capable of generating some wild-type transcripts exhibit remarkable differences not only between minigene and full-length gene splicing assays but also between different minigene assays. Our results therefore bring fresh glimpses of the limitations that are inherent to minigene splicing assays and emphasize the role of sequence context in regulating splicing of a particular variant type. Whether our findings also apply to other types of splice-altering variant remains to be investigated.

## Data Availability Statement

The original contributions presented in the study are included in the article/Supplementary Material, further inquiries can be directed to the corresponding author/s.

## Author Contributions

J-HL, HW, and W-BZ designed the study, performed the experiments, and assisted in writing the manuscript. EM, YF, GLG, DNC, and CF analyzed the data and revised the manuscript with important intellectual input. ZL contributed to study design, obtained funding, supervised the experiments, and revised the manuscript with important intellectual input. J-MC conceived and coordinated the study and drafted the manuscript. All authors approved the final manuscript.

## Conflict of Interest

The authors declare that the research was conducted in the absence of any commercial or financial relationships that could be construed as a potential conflict of interest.

## Publisher’s Note

All claims expressed in this article are solely those of the authors and do not necessarily represent those of their affiliated organizations, or those of the publisher, the editors and the reviewers. Any product that may be evaluated in this article, or claim that may be made by its manufacturer, is not guaranteed or endorsed by the publisher.

## Footnotes

1
https://science.umd.edu/labs/mount/RNAinfo/consensus.html

2
https://www.ncbi.nlm.nih.gov/gene/6232
