# Supplementary material for: Splicing Outcomes of 5′ Splice Site GT>GC Variants That Generate Wild-Type Transcripts Differ Significantly Between Full-Length and Minigene Splicing Assays
Source: Front Genet. 2021 Aug 5;12:701652. doi: 10.3389/fgene.2021.701652 (PMC8375439; doi:10.3389/fgene.2021.701652)
Supplement: Supplementary file 1 [file Data_Sheet_1.PDF]

Splicing outcomes of 5' splice site GT>GC variants that generate wild-type transcripts differ significantly between full-length and minigene splicing assays.

Jin-Huan Lin, Hao Wu, Wen-Bin Zou, Emmanuelle Masson, Yann Fichou, Gerald Le Gac, David N. Cooper, Claude Férec, Zhuan Liao, Jian-Min Chen

Supplementary Table S1. Additional information about the 20 +2T>C variants analyzed in this study

Gene	mRNA reference	Variant	Chromosome	hg38		HGVS nomenclature
				Coordinate	Reference sequence	
CD3E	NM_000733.3	IVS7+2T>C	11	118313876	T	c.520+2T>C
CD40LG	NM_000074.2	IVS3+2T>C	X	136654432	T	c.346+2T>C
DBI	NM_001079862.2	IVS2+2T>C	2	119368307	T	c.127+2T>C
DMD	NM_004006.2	IVS54+2T>C	X	31657988	A	c.8027+2T>C
DNAJC19	NM_145261.3	IVS5+2T>C	3	180985924	A	c.280+2T>C
FOLR3	NM_000804.3	IVS4+2T>C	11	72139484	T	c.493+2T>C
HBB	NM_000518.5	IVS2+2T>C	11	5226575	A	c.315+2T>C
IFNL2	NM_172138.1	IVS5+2T>C	19	39269823	T	c.504+2T>C
IL10	NM_000572.3	IVS3+2T>C	1	206770905	A	c.378+2T>C
MGP	NM_000900.4	IVS2+2T>C	12	14884211	A	c.94+2T>C
PLP1	NM_000533.4	IVS5+2T>C	X	103788512	T	c.696+2T>C
PSMC5	NM_001199163.1	IVS6+2T>C	17	63830503	T	c.528+2T>C
		IVS8+2T>C	17	63831228	T	c.846+2T>C
		IVS10+2T>C	17	63831618	T	c.1056+2T>C
RPL11	NM_000975.5	IVS2+2T>C	1	23692761	T	c.157+2T>C
		IVS3+2T>C	1	23693915	T	c.264+2T>C
RPS27	NM_001030.4	IVS2+2T>C	1	153991225	T	c.115+2T>C
		IVS3+2T>C	1	153991678	T	c.226+2T>C
SELENOS	NM_203472.2	IVS5+2T>C	15	101274418	A	c.484+2T>C
SPINK1	NM_003122.3	IVS3+2T>C	5	147828020	A	c.194+2T>C

Supplementary Table S2. Wild-type genomic sequences inserted into the pET01 and pSPL3 exon trapping vectors

Gene	Variant	Corresponding wild-type genomic sequences cloned into both pET01 and pSPL3 vector[a]	Size (bp)
CD3E	IVS7+2T>C	gggatgaggaaacagagggtttgtgagttgaaaacacatttcacgcttctcacagctagtgagtaataaagctgggactcaaacccagggctgtttgactccagtgc<br>ctctacccacggccaccactctttgcttgtcaatgttgttctaaacatattgaaggggggctctgaccgtggcaagcgtgtgagtagtaaggggagaatggccttcat<br>gcactccctcctcacctccagcgccttgtgttttccttgcttagtgatttcccctctccccaccccaccccccacagTGTGTGAGAACTGCATGGAG<br>ATGGATGTGATGTCGGTGGCCACAATTGTCATAGTGGACATCTGCATCACTGGGGGCTTGCTGCTG<br>CTGGTTTACTACTGGAGCAAGAATAGAAAGGCCAAGGCCAAGCCTGTGACACGAGGAGCGGGTGC<br>TGGCGGCAGGCAAAGGGgtaaggctgtggagtccagtcagaggagattcctgccaagggggacgaccagcctgggccagggtgggtggcaag<br>tccacagctaggtcagaacagcttctctagagcttctatgcacagcttctattactgtgatgacaagatctcaacagacggtttcaaatctcacatcactcccctccttcc<br>catcctagaaaagtgcaaaaaagtttatgaaagtgatgggcttcctcacatacctgtcaatgcctgcagtcatccgattccgcccctaagctgtgggaagagagact	754
CD40LG	IVS3+2T>C	gaggtcataggaagggtcaggatcacagcctctggtctggagagagcactggaatggagataataaggcctggattttacttccagattctcccctgggctttctggg<br>ttgttggctcatctgtcagatccatggactcccaattggcatgatggaattaatgacaggatctgagtctatatgataatcctcaccagaaacagacaacagagtaatga<br>cagatgcaaaacgaatgataattttaaaccccacagcagagcccctgtcaaaatgacctcttgcaatgcttcttattttagGATATAATGTTAAACAA<br>AGAGGAGACGAAGAAAGAAAACAGCTTTGAAATGCAAAAAGgtaggtttgctatttgctaatttctatgaatgcctaaaaact<br>aaaaggaagctttaggctgatcatattgaacaacccagtgttgttgcatcagggaacttttagccctggaaataaaacaggaacacaattgtcaaattgacaccttctct<br>ggtccctgtgatttggaaagactttgtacatatatatttatgaaaaaaggatgtgttcctttaatgccgatgataccaaatctgaagaaatcccattatgttcaataccttaat<br>agaagcaaccatacagcctga	645
DBI	IVS2+2T>C	tgccctcttcactgctgtatttccagacctgatgcctgcgtttgtgagagctctggatatatggttttcgattgaatgagtgaactggaggggcttccccttcttgtgttgct<br>gaatctttctagctgccctgttggggcagggaggggcagacacacttcaggggctgcattgcccgaagggtgccacctttcccacctctccatccccgtaactggg<br>ctgtcatcaggccacagtaggattcttaccctctcccacccagaggaggccctcaatcctctcctctcccttccatttagGCTGAGTTTGAGAAAGCT<br>GCAGAGGAGGTTAGGCACCTTAAGACCAAGCCATCGGATGAGGAGATGCTGTTCATCTATGGCCAC<br>TACAAACAAGCAACTGTGGGCGACATAAATACAGgtatgcagagcgggggttggaagggcatctgctcatcaaagcaggctcag<br>cagctcagactggaagtccctgggaacttcactctcaaactgcctgaggccctactcttcaggtggggtatggtgatggttcctgaggtggaaaagaccatgttccg<br>gattctcagtgtctccagtagtaacagaattcaaatcctggttttagaaggtctttactggttatcaccagcagctactctctactagggaagaagcaaaggctgcagctt<br>ggaaaagacttgc	698
DMD	IVS54+2T>C	tgtgataaaaacaattaagcccactttattgccaattaattgctactaagttgaaatacttgatactggttattgctcaagatgctgcatttgaaaagtttgtcctgaaaggtg<br>ggttaccttatactgtcatgattgactaaatcatatggtaggttaaaagcaatctaatatatgtattctgacctgaggattcagaagctgtttacgaagtattttaagacactc<br>caactagagatttcataaaaaaaactgacattcattctctttctcataaaaatctatagCAGTTGGCCAAAGACCTCCGCCAGTGGCAGAC<br>AAATGTAGATGTGGCAAATGACTTGGCCCTGAAACTTCTCCGGGATTATTCTGCAGATGATACCAG<br>AAAAGTCCACATGATAACAGAGAATATCAATGCCTCTTGGAGAAGCATTCATAAAAGgtatgaattacattat<br>ttctaaaactactgttggctgtaataatggggtggtgaaactggatggaccatgaggatttgtttttccaatccagctaaactggagcttgggagggttcaagacgataa<br>ataccaactaaactcacggacttggctcagacttctattttaaaaacgaggaacataagatctcatttgcccgctgtcacaaaagtagtgacataaccaagagattaaa<br>caaaaagcaaaatactgatttatagctagaagagccatttatcagtct	725

DNAJC19	IVS5+2T>C	ctaggggacagagtgagactatctcaaaaaaaaaaaaaaaacaaaactcttccttcttcattttcaaatccttaaacatgcgtttcttatcaataaaaggactgttgtaatgggtactgcaagttcaacacatttttgagagtgccttacttttctaagtagtttcccaatttaggatacgcattgggtgtgttgaggtttctaaacagcttttcatactgttgtgttaatttactacagtttcacagtgtctttaattggcctttatgtacagtttttttgatgtgatgcagaagtaggatgaagtaaataccatttctttttttaattagCCCTACTGCCAATAAAGGGAAAATAAGAGATGCTCATCGACGAATTATGCTTTTAAATCATCCTGACAAAGgtaagtagtcattaaattctcgttgatgttgttggtgtgaccaatagttattaatgggataattttatctcaaattcctggctattagaaaggtaacacttaaatctgtaagtcctgtctcctaggctggagccatatatttttggtgtcttaaaagaaattaagtgaaaactttacttacagaatgagcagatgtaagtgctactattactatgttttgtgggtttttctggcctacatttattataattaaaacgttaaatttcgtaaaactaaaatgtaatttttccttatccattttagataccttgaaacggagattacctag	729
FOLR3	IVS4+2T>C	gttcccacaaacattaacctcagcagagggcggagcctgccagttgctggcagggagggcttggtccaggaattcgggtctgagggtggtggacgccctgccccctcccacagctctggtccccttcaagggtaaagctgctgagatacgtggctgacaggagtattctgtctcctccccactcagGTCAACCAGAGCTGGCGCAAAGAGCGCATTCTGAACGTGCCCCTGTGCAAAGAGGACTGTGAGCGCTGGTGGGAGGACTGTCGCACCTCCTACACCTGCAAAAGCAACTGGCACAAAGGCTGGAATTGGACCTCAGgtgaggacctgaggagataagatgaggagtgggagtggggctttggggttgggaggggtgcggtctggcccagaagctaagg	404
HBB	IVS2+2T>C	ttggtatcaaggttacaagacaggtttaaggagaccaatagaaactgggcatgtggagacagagaagactcttgggtttctgataggcactgactctctctgcctattggtctattttcccacccttagGCTGCTGGTGGTCTACCCTTGGACCCAGAGGTTCTTTGAGTCCTTTGGGGATCTGTCCACTCCTGATGCTGTTATGGGCAACCCTAAGGTGAAGGCTCATGGCAAGAAAGTGCTCGGTGCCTTTAGTGATGGCCTGGCTCACCTGGACAACCTCAAGGGCACCTTTGCCACACTGAGTGAGCTGCACTGTGACAAGCTGCACGTGGATCCTGAGAACTTCAGGgtgagtctatgggacgcttgatgttttctttccccttcttttctatggttaagttcatgtcataggaaggggataagtaacagggtacagtttagaatgggaaacagacgaatgattgcatcagtgtggaagtctcaggatcgttttagtttcttttatttgc	515
IFNL2	IVS5+2T>C	tctgagcagcgtccttccccttgccaaggccccggctcacacaccgccctcctctgcccacagATCCAGCCTCAGCCCACGGCAGGGCCCAGGACCCGGGGCCGCCTCCACCATTGGCTGTACCGGCTCCAGGAGGCCCCAAAAAAGgtgagtgacccgggaagagagggactgaggtctggggagccactgggagcccagaacccagacag	212
IL10	IVS3+2T>C	agcatgattaagggaagggagactctgcttcctgattgcagggaattgggtttgtttccttcgctttgaaaaggagaagtgggaagatgttaactcagcacatccagcagccagagggtttacaaagggctcagtcccttcggggaggcttctggtgaaggaggatcgctagaaccaagctgtcctcttaagctagttgcagcagcccctcctcccagccacctccgccaatctctcactcacctttggctcctgcccttagGGTTACCTGGGTTGCCAAGCCTTGTCTGAGATGATCCAGTTTTACCTGGAGGAGGTGATGCCCCAAGCTGAGAACCAAGACCCAGACATCAAGGCGCATGTGAACTCCCTGGGGGAGAACCTGAAGACCCTCAGGCTGAGGCTACGGCGCTGTgtaagtagcagatcagtttttttcccttgcagctgcccccaaaataccatctcctacagaccagcagggacactcacatccacagacacagcaaagacacagactggcagagctagctgtaaatgaggaaagactcctggagtcagatctcttgctcatttctctttgagcaggcgttgggggtggctgctaggcatttacatgtgaaatttgcaaacagctttcctgttatttgtgagtcatttgtgggttattaactactcccctctctcttcataaaaggagcccagagcttcagtcaggcct	720
MGP	IVS2+2T>C	acgtcatagcccttgggaaattttagctgaaaatggccactccctccttcaacatcagagaaactaaaatatagagatatccacagcaaggccagagctagagaaaaacctcataaatcctaaattcctgaaatttctaataaccacactgctaaatatattcttcatgtttttagactctttcctcttcttccatccctgtatttaaactatcacagtgtctaaattgataaataataacataatgaatcatggataaatattgatataatgaatcttttttttttaatttcagAATCACATGAAAGCATGGAATCTTATGAACTTAgtaagtgaatatttaacttctttattcaaatcccttgcattaaagaacctcttcttatttttaaataaacaagatggaaagatatataacagggagggaaaagggggcctcttttggaaaactaaagtaaatttttaaatctaatgactataaaaattgccaaaggagcaatttttttaagtttgaagtagtgcaatatgggatttaagctacaggcgacatatttagaagccataaaatctcatttggaaattttaaattggcaccacgtcaactg	604

PLP1	IVS5+2T>C	gccattcacattggcctactctagttgactgctgttcacaaccccaaagcagcacatttcaataacaaacacaaggtttcaccactgttcaataccaccttctctttttgta<br>aacctgtagaaaagaggatcctgattgttggtagaatccaactttacagccaggataattagagatggaagaagggctctgggggaaagtctccatgtggccccgta<br>actccataaagcttaccctgcttgctttttgtgtcttacttagGTGTTCTCCCATGGAATGCTTTCCCTGGCAAGGTTTGTGGCTC<br>CAACCTTCTGTCCATCTGCAAAACAGCTGAGgtgagtgggttatttgggttattttacaagggagtagctaataccatacaaattacaccc<br>atggccttcaattttaaggactgaaagtttccctttgctggattttgaattagccgattgccttctacaacatgttggctaagtgtgcctgagccaatgagcatagaaggta<br>aaacacctgttttctctagagttgcatagaaagacttcctttccaacccttcccctcttaaaagagaagtttgtagctttaggtaaagatagagcctaatggcaaaatccc<br>ccacatgaaaa	631
PSMC5	IVS6+2T>C	caggtgaggtggtggtggtggggtcagctcttactgtaccacttctgaaactcgccccccttcacccagGTGACACCCAATTGCCGGGTGG<br>CTCTAAGGAATGACAGCTACACTCTGCACAAGATCCTGCCCAACAAGGTAGACCCATTAGTGTCAC<br>TGATGATGGTGGAGAAAGTACCAGATTCAACTTATGAGATGATTGGTGGACTGGACAAACAGATC<br>AAGGAGATCAAAGAAGTGATCGAGCTGCCTGTTAAGCATCCTGAGCTCTTCGAAGCACTGGGCATT<br>GCTCAGCCCAAGgtgaggagcagggcttctctgagagggccaagctgtacttactcctcctgccccagccagccctactgcaggggttgggg	382
	IVS8+2T>C	tgagaagcaagaggtaggggtaggggggttagagagctaataagctaataagctccctaacaccagctcggcctccacacagGGGCAAGAATGGTG<br>AGGGAGCTGTTTGTCATGGCACGGGAACATGCTCCATCTATCATCTTCATGGACGAAATCGACTCC<br>ATCGGCTCCTCGCGGCTGGAGGGGGGTTCTGGAGGGGACAGTGAAGTGCAGCGCACGATGCTGGA<br>GTTGCTCAACCAGCTCGACGGCTTTGAGGCCACCAAGAACATCAAGgtaaggtggtagcatccttgggatgggcccag<br>ggaaggcctgggtgccacgcaggctgaggaagaggtttagctgatccccacttgct	360
	IVS10+2T>C	gcaggctgaggaagaggtttagctgatccccacttgctttctctgctcagGTTATCATGGCTACTAATAGGATTGATATCCTGGAC<br>TCGGCACTGCTTCGCCCAGGGCGCATTGACAGAAAAATTGAATTCCCACCCCCCAATGAGGAGgtttgt<br>gatggacactgtgcaaagtggctctggctgtgggggtggggtgtggggctcaggcttttccttgccatctccagGCCCGGCTGGACATTTTGAA<br>GATTCATTCTCGGAAGATGAACCTGACCCGGGGGATCAACCTGAGAAAAATTGCTGAGCTCATGCC<br>AGGAGCATCAGGGGCTGAAGTGAAGgtaattggagtacccactgaaaacagggcagaggcaggaagctctgggctcaagggccacaga<br>tgaggggcacagcagtggggcctcaatttcctt	436
RPL11	IVS2+2T>C	gcgctctttgttaccctgagcctcttagggttcgatctcagtgtccgtatcttttaacactcaataactgtcctgagttttctcttcacccgttccgttcgtggaggaaggata<br>ggttccgagctgtcttcttcccttgatgtcccctaaacattataccttttaaacattcagggtcttcgttacgatttgggatgagcagaaataaaaatgctgtgcagataga<br>aagtagtaaaactcagggccctcagctgtgagtgtattgactgctgctcttccctgttgcagCAGGATCAAGGTGAAAAGGAGAACCCCA<br>TGCGGGAACTTCGCATCCGCAAACTCTGTCTCAACATCTGTGTTGGGGAGAGTGGAGACAGACTGA<br>CGCGAGCAGCCAAGGTGTTGGAGCAGCTCACAGGGCAGACCCCTGTGTTTTCCAAAGgtgagtagtcacaa<br>ggacatacagggtttgcctgcttgggtcgcttggttgtttcttgatttacctgctgtcgagtctgtttagaaagtgacagtcggcatcacttaaagcattaaattcatgagc<br>cggccaagaggtgtcttttttttttttttttattcaagatggcgtgtgggattggaacactagattttatttgagcagatcttaagctaagactagcccaagtaaagattttcct<br>aagtaactaggatatgagatagagtggaaatgtcaggaacga	722
	IVS3+2T>C	tcggcctattctggttgtgaccttgcccttcctggaacttcggcgttatgactgttcttaactgctgaaggatggctggatgtctggaaatgggaaaatctgtgctgtgga<br>tgaaatcttattaatagatgtgggagacactaattagaacaccacaacttaaaagagtgtggatgaatgcttaatgtctctttaagtcatggagatggtgttctgggaaag<br>aggtgagtgtagtgggggtatgatggcatctgactccttgttacccacttcctgcagCTAGATACACTGTCAGATCCTTTGGCATCCGG<br>AGAAATGAAAAGATTGCTGTCCACTGCACAGTTCGAGGGGCCAAGGCAGAAGAAATCTTGGAGAA	656

		GGGTCTAAAGgtgagcctaatcccctaatggagtgatattgatcagcactcctttagtaacacatgtagataagttacatttaatgttctgttctttggtgtcttgat<br>atttatttacttaagcttctaaaaggctttttctacaatcagcagggttaaactgttcttggtggtttaaaagatgcttgaggctgggcacggtggctcaacgcctgtaatcc<br>caacactttgggaggccaaggcggttggatcatttgtggccaggagttcgagaccatcc	
RPS27	IVS2+2T>C	gcacttcttaggacattaactccagggaccgcagcggcccacgggccacccgcatagacgggagcggagaggagataagatggcggcccagctgcgcagaca<br>ccaggggcggcgaggggcgagctctccccggtgtgtgacagtgggggctattttcgaccatcccattttcgctgttggtttctaaatctctgcatttctgtccctcttag<br>CTCGCAAAGGATCTCCTTCATCCCTCTCCAGAAGAGGAGAAGAGGAAACACAAGAAGAAACGCCT<br>GGTGCAGAGCCCCAATTCCTACTTCATGGATGTGAAATGCCCAGgtgaggagacggcttgctgtagtggggaaagcact<br>ggacctcaacagttggaaaatgttgtagtgttagctgtctcgtatccttgaagctgtgcagcagcttcagtttcttcgcctgtggaaaatattttccctgatactcttaaaatt<br>tgaatgtatgagactggcaaagttttgcatcttaggaggagtgattcatttcaccgtgatctctca	536
	IVS3+2T>C	agtttcttcgcctgtggaaaatattttccctgatactcttaaaatttgaatgtatgagactggcaaagttttgcatcttaggaggagtgattcatttcaccgtgatctctcatca<br>catttcacatacaacccctacgtttttttgtgttgggaaacaatgtaatggatgatgagttgggcataagtgcaggaaagacgggtgtaatagaggaaaaaaatgttatc<br>tgcttttctttcagGATGCTATAAAATCACCACGGTCTTTAGCCATGCACAAACGGTAGTTTTGTGTGTTGGC<br>TGCTCCACTGTCCTCTGCCAGCCTACAGGAGGAAAAGCAAGGCTTACAGAAGgtaaatggtttactaatgtgatttg<br>gggctttgagtttgatttttagaaatggaaacatttcttaggatttttcggtcttaacagtgacagggatcatctataatgtaaatttttagacaagaagtgttggatttggttg<br>ttttaactagatactaccaaaagctatgtattaatctcaaagcactactgtctaaagcggggataaatgggtgataaaagggcttaagttga	582
SELENOS	IVS5+2T>C	acgagggtgaagtttgttctgaaagacatttaaattaagaattatcagagttagctttgtctttgagagaaatggcagcttctgaattctttctgtaaaatgtgattgtttctca<br>gCTTGAAGAAGAAAAAAGGAGACAGAAGATTGAAATGTGGACAGCATGCAAGAAGGAAAAAGT<br>TACAAAGGAAATGCAAAGAAGCCCCAGgtgactggagacctcggccggctggcatgcggtagatgaagattgccaagtagaatgtttta<br>attgcttcttacactactgtgtgtgttcaaacagGAGGAAGACAGTCCTGGGCCTTCCACTTCATCTGTCCTGAAACGGAA<br>ATCGGACAGAAAGCCTTTGCGGGGAGGAGgtaagcaccactgatgtcaaatgttaacagattttcaacacttacaggatatagttaccttttt<br>aggaacaagattgtttgtttctttgtccataaattaagactaattccttaggattgtgaagattcaataaaggaaacagatgcaaatcacctcctaggtcctcactaagtac<br>ttagaaggattgtacttatagtattctaacttgatccttctgcagccccgtagagggagagctaagtagggtgaggaattgtctgccaatcttcagatgagtgtcaagga<br>gctggaaca	672
SPINK1	IVS3+2T>C	tttcagaagggccataggacttactaatgtcacacagcttagaaatagcagaggcatgacttaaaacaaggttttctgtctccagatagtaggttatttctcttacaacac<br>acagtatcattctcccaatcacagttattccccagagaaataaaaccatttcagagattttgctatgaactcaagaatggagaataatgggaaatgattctgtttaattcca<br>tttttagGCCAAATGTTACAATGAACTTAATGGATGCACCAAGATATATGACCCTGTCTGTGGGACTGAT<br>GGAAATACTTATCCCAATGAATGCGTGTTATGTTTTGAAAATCGgtgagtacaaacttgagtttcttttaaactatatattttaa<br>gttagttatcttcaagtgtactgataatatgaatctcaccccgagaaaagcaaactatttactttttccaaaaacagttatctctttcttattctcccttttatatatttagcattaa<br>atattatttttttagaagtcacttgtatgataaaagcctatatttttttacagcaaaatagtcgatagcttgg	567

[a] Sequences of exon N (or exon N-1 and exon N; see Figure 1 for definition) are in uppercase letters.

Supplementary Table S3. In-fusion cloning primers used in this study

Gene	Variant	Vector	Primer sequence (5' > 3')[a]	Location
CD3E	IVS7+2T>C	pET01	Forward: CGGGCCCCCCTCGAGgggatgaggaaacagagggt<br>Reverse: TAGAACTAGTGGATCCagtctctcttcccacagctt	Intron 6<br>Intron 7
		pSPL3	Forward: AATTCTGGAGCTCGAGgggatgaggaaacagagggt<br>Reverse: CAGATATCTGGGATCCagtctctcttcccacagctt	Intron 6<br>Intron 7
CD40LG	IVS3+2T>C	pET01	Forward: CGGGCCCCCCTCGAGgaggtcataggaagggtcagg<br>Reverse: TAGAACTAGTGGATCCtcaggctgtatggttgcttc	Intron 2<br>Intron 3
		pSPL3	Forward: AATTCTGGAGCTCGAGgaggtcataggaagggtcagg<br>Reverse: CAGATATCTGGGATCCtcaggctgtatggttgcttc	Intron 2<br>Intron 3
DBI	IVS2+2T>C	pET01	Forward: CGGGCCCCCCTCGAGtgccctcttcactgctgtat<br>Reverse: TAGAACTAGTGGATCCgcaagtcttttccaagctgc	Intron 1<br>Intron 2
		pSPL3	Forward: AATTCTGGAGCTCGAGtgccctcttcactgctgtat<br>Reverse: CAGATATCTGGGATCCgcaagtcttttccaagctgc	Intron 1<br>Intron 2
DMD	IVS54+2T>C	pET01	Forward : CGGGCCCCCCTCGAGtgtgataaaaacaattaagcccact<br>Reverse : TAGAACTAGTGGATCCagactgataaatggctcttctagc	Intron 53<br>Intron 54
		pSPL3	Forward : AATTCTGGAGCTCGAGtgtgataaaaacaattaagcccact<br>Reverse : CAGATATCTGGGATCCagactgataaatggctcttctagc	Intron 53<br>Intron 54
DNAJC19	IVS5+2T>C	pET01	Forward: CGGGCCCCCCTCGAGctaggggacagagtgagact<br>Reverse: TAGAACTAGTGGATCCctaggtaatctccgtttcaaggt	Intron 4<br>Intron 5
		pSPL3	Forward: AATTCTGGAGCTCGAGctaggggacagagtgagact<br>Reverse: CAGATATCTGGGATCCctaggtaatctccgtttcaaggt	Intron 4<br>Intron 5
FOLR3	IVS4+2T>C	pET01	Forward : CGGGCCCCCCTCGAGgttcccacaaacattaacctcag<br>Reverse : TAGAACTAGTGGATCCccttagcttctgggccag	Intron 3<br>Intron 4
		pSPL3	Forward : AATTCTGGAGCTCGAGgttcccacaaacattaacctcag<br>Reverse : CAGATATCTGGGATCCccttagcttctgggccag	Intron 3<br>Intron 4
HBB	IVS2+2T>C	pET01	Forward : CGGGCCCCCCTCGAGttggtatcaaggttacaagacagg<br>Reverse : TAGAACTAGTGGATCCgcaaataaaagaaactaaaacgatcc	Intron 1<br>Intron 2
		pSPL3	Forward : AATTCTGGAGCTCGAGttggtatcaaggttacaagacagg<br>Reverse : CAGATATCTGGGATCCgcaaataaaagaaactaaaacgatcc	Intron 1<br>Intron 2
IFNL2	IVS5+2T>C	pET01	Forward : CGGGCCCCCCTCGAGtctgagcagcgtccttcc<br>Reverse : TAGAACTAGTGGATCCctgtctgggttctgggctc	Intron 4<br>Intron 5
		pSPL3	Forward : AATTCTGGAGCTCGAGtctgagcagcgtccttcc	Intron 4

			Reverse : CAGATATCTGGGATCCctgtctgggttctgggctc	Intron 5
IL10	IVS3+2T>C	pET01	Forward : CGGGCCCCCCTCGAGagcatgattaagggaagggaga<br>Reverse : TAGAACTAGTGGATCCaggcctgactgaagctctg	Intron 2<br>Intron 3
		pSPL3	Forward : AATTCTGGAGCTCGAGagcatgattaagggaagggaga<br>Reverse : CAGATATCTGGGATCCaggcctgactgaagctctg	Intron 2<br>Intron 3
MGP	IVS2+2T>C	pET01	Forward : CGGGCCCCCCTCGAGacgtcatagcccttgggaaa<br>Reverse : TAGAACTAGTGGATCCcagttgacgtggtgccaatt	Intron 1<br>Intron 2
		pSPL3	Forward : AATTCTGGAGCTCGAGacgtcatagcccttgggaaa<br>Reverse : CAGATATCTGGGATCCcagttgacgtggtgccaatt	Intron 1<br>Intron 2
PLP1	IVS5+2T>C	pET01	Forward : CGGGCCCCCCTCGAGgccattcacattggcctact<br>Reverse : TAGAACTAGTGGATCCttttcatgtgggggattttg	Intron 4<br>Intron 5
		pSPL3	Forward : AATTCTGGAGCTCGAGgccattcacattggcctact<br>Reverse : CAGATATCTGGGATCCttttcatgtgggggattttg	Intron 4<br>Intron 5
PSMC5	IVS6+2T>C	pET01	Forward : CGGGCCCCCCTCGAGcaggtgaggtggtggtgg<br>Reverse : TAGAACTAGTGGATCCccccaacccctgcagtag	Intron 5<br>Intron 6
		pSPL3	Forward : AATTCTGGAGCTCGAGcaggtgaggtggtggtgg<br>Reverse : CAGATATCTGGGATCCccccaacccctgcagtag	Intron 5<br>Intron 6
	IVS8+2T>C	pET01	Forward : CGGGCCCCCCTCGAGtgagaagcaagaggtagggg<br>Reverse : TAGAACTAGTGGATCCagcaagtggggatcagctaa	Intron 7<br>Intron 8
		pSPL3	Forward : AATTCTGGAGCTCGAGtgagaagcaagaggtagggg<br>Reverse : CAGATATCTGGGATCCagcaagtggggatcagctaa	Intron 7<br>Intron 8
	IVS10+2T>C	pET01	Forward : CGGGCCCCCCTCGAGgcaggctgaggaagaggttt<br>Reverse : TAGAACTAGTGGATCCaaggaaattgaggccccact	Intron 8<br>Intron 10
		pSPL3	Forward : AATTCTGGAGCTCGAGgcaggctgaggaagaggttt<br>Reverse : CAGATATCTGGGATCCaaggaaattgaggccccact	Intron 8<br>Intron 10
RPL11	IVS2+2T>C	pET01	Forward : CGGGCCCCCCTCGAGgcgctctttgttaccctgag<br>Reverse : TAGAACTAGTGGATCCtcgttcctgacatttccactc	Intron 1<br>Intron 2
		pSPL3	Forward : AATTCTGGAGCTCGAGgcgctctttgttaccctgag<br>Reverse : CAGATATCTGGGATCCtcgttcctgacatttccactc	Intron 1<br>Intron 2
	IVS3+2T>C	pET01	Forward : CGGGCCCCCCTCGAGtcggcctattctggttgtga<br>Reverse : TAGAACTAGTGGATCCggatggtctcgaactcctgg	Intron 2<br>Intron 3
		pSPL3	Forward : AATTCTGGAGCTCGAGtcggcctattctggttgtga<br>Reverse : CAGATATCTGGGATCCggatggtctcgaactcctgg	Intron 2<br>Intron 3
RPS27	IVS2+2T>C	pET01	Forward : CGGGCCCCCCTCGAGgcacttcttaggacattaactcca<br>Reverse : TAGAACTAGTGGATCCtgagagatcacggtgaaatga	Intron 1<br>Intron 2

		pSPL3	Forward : AATTCTGGAGCTCGAGgcacttcttaggacattaactcca<br>Reverse : CAGATATCTGGGATCCtgagagatcacggtgaaatga	Intron 1<br>Intron 2
	IVS3+2T>C	pET01	Forward : CGGGCCCCCCCTCGAGagtttcttcgcctgtggaaa<br>Reverse : TAGAACTAGTGGATCCtcaacttaagcccttttatcacc	Intron 2<br>Intron 3
		pSPL3	Forward : AATTCTGGAGCTCGAGagtttcttcgcctgtggaaa<br>Reverse : CAGATATCTGGGATCCtcaacttaagcccttttatcacc	Intron 2<br>Intron 3
SELENOS	IVS5+2T>C	pET01	Forward : CGGGCCCCCCCTCGAGacgagggtgaagtttgttctg<br>Reverse : TAGAACTAGTGGATCCtgttccagctccttgacact	Intron 3<br>Intron 5
		pSPL3	Forward : AATTCTGGAGCTCGAGacgagggtgaagtttgttctg<br>Reverse : CAGATATCTGGGATCCtgttccagctccttgacact	Intron 3<br>Intron 5
SPINK1	IVS3+2T>C	pET01	Forward : CGGGCCCCCCCTCGAGtttcagaagggccataggac<br>Reverse : TAGAACTAGTGGATCCccaagctatcgactattttgctg	Intron 2<br>Intron 3
		pSPL3	Forward : AATTCTGGAGCTCGAGtttcagaagggccataggac<br>Reverse : CAGATATCTGGGATCCccaagctatcgactattttgctg	Intron 2<br>Intron 3

[a] Sequences homologous to the linearized vector end are in uppercase letters, with the *Xho*I or *BamH*I restriction site being highlighted in blue and red, respectively. Gene-specific sequences are in lowercase letters.

Supplementary Table S4. Primers used for site-directed mutagenesis

Gene	Variant	Primer sequence (5' > 3')[a]
CD3E	IVS7+2T>C	aggcaaaggggCaaggctgtggagt
CD40LG	IVS3+2T>C	gctttgaaatgcaaaaaggCaggtttgctatttgc
DBI	IVS2+2T>C	gacataaatacaggCatgcagagcgggg
DMD	IVS54+2T>C	ggagaagcattcataaaaggCatgaattacattatttc
DNAJC19	IVS5+2T>C	catcctgacaaaggCaagtagtcattaaattctcg
FOLR3	IVS4+2T>C	gaattggacctcaggCgaggacctgaggag
HBB	IVS2+2T>C	gagaacttcagggCgagtctatgggac
IFNL2	IVS5+2T>C	cccaaaaaaggCgagtgacccgggaag
IL10	IVS3+2T>C	ctacggcgctgtgCaagtagcagatc
MGP	IVS2+2T>C	catggaatcttatgaacttagCaagtgaatatttaacttc
PLP1	IVS5+2T>C	caaaacagctgaggCgagtgggttatttgg
PSMC5	IVS6+2T>C	attgctcagcccaaggCgaggagcagggcttctct
	IVS8+2T>C	caagaacatcaaggCaaggtggtagcatcc
	IVS10+2T>C	ggctgaagtgaaggCaattggagtaccc
RPL11	IVS2+2T>C	ctgtgttttccaaaggCgagtagtcacaaggac
	IVS3+2T>C	gaagggtctaaaggCgagcctaatcccc
RPS27	IVS2+2T>C	gatgtgaaatgcccaggCgaggagacggcttgctg
	IVS3+2T>C	gcaaggcttacagaaggCaaatggtttactaatg
SELENOS	IVS5+2T>C	cggggaggaggcaagCaccactgatgtcaaatg
SPINK1	IVS3+2T>C	gttttgaaaatcggCgagtacaaacttgag

[a]Only the forward primer is shown. The variant introduced is in uppercase and highlighted in red.

pET01 minigene

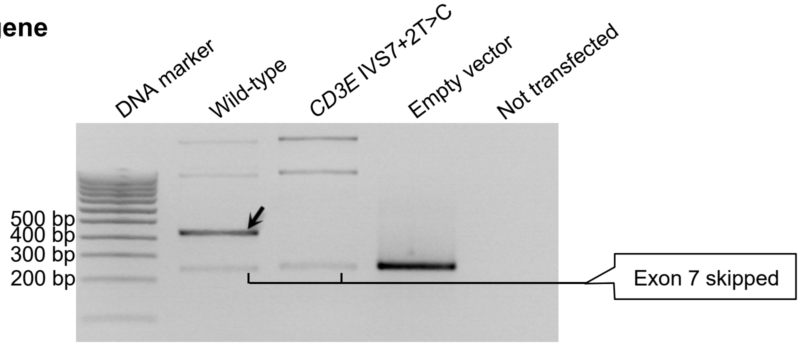

pSPL3 minigene

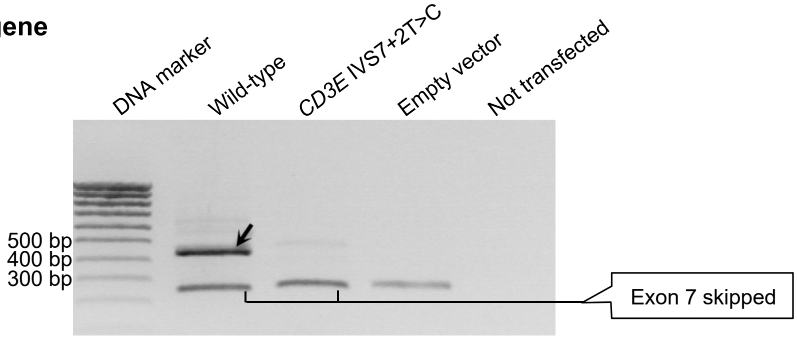

Supplementary Figure S1. Reverse transcription-polymerase chain reaction results from the pET01 and pSPL3 minigene assays with respect to the *CD3E* IVS7+2T>C variant. In all panels, wild-type transcripts are indicated by oblique downward pointing arrows.

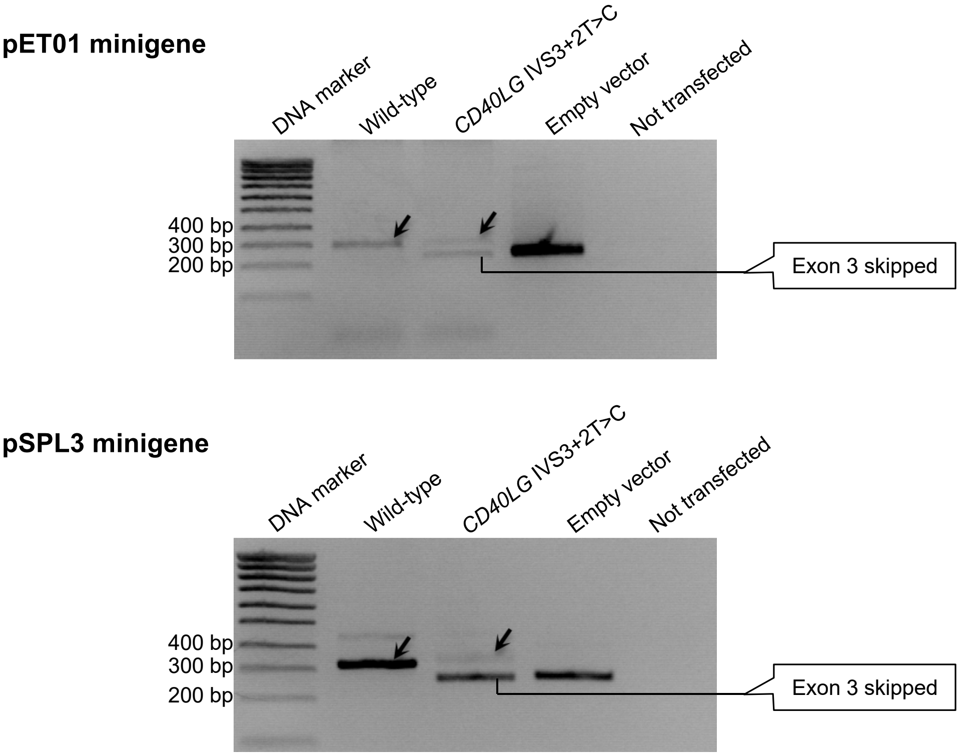

Supplementary Figure S2. Reverse transcription-polymerase chain reaction results from the pET01 and pSPL3 minigene assays with respect to the *CD40LG* IVS3+2T>C variant. In all panels, wild-type transcripts are indicated by oblique downward pointing arrows.

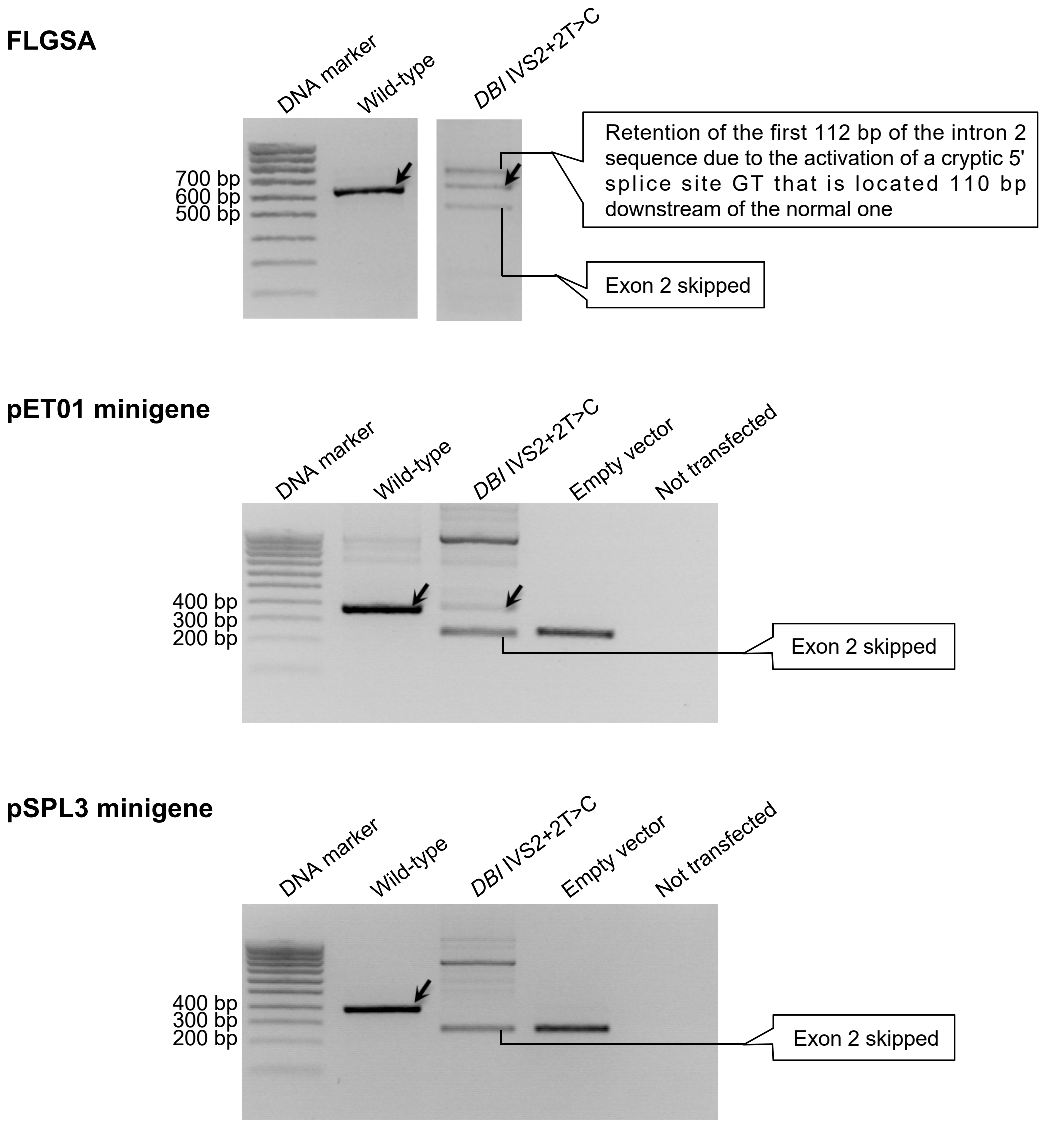

Supplementary Figure S3. Reverse transcription-polymerase chain reaction (RT-PCR) results from the pET01 and pSPL3 minigene assays with respect to the *DBI* IVS2+2T>C variant. Results from the previously performed full-length gene splicing assay (FLGSA) [12] are included for the sake of comparison (NB. The two aberrant transcripts were newly sequenced in this study). In all panels, wild-type transcripts are indicated by oblique downward pointing arrows. The FLGSA data were adapted from Lin et al. (2019) [12] with permission (Copyright 2020 Wiley Periodicals LLC).

pET01 minigene

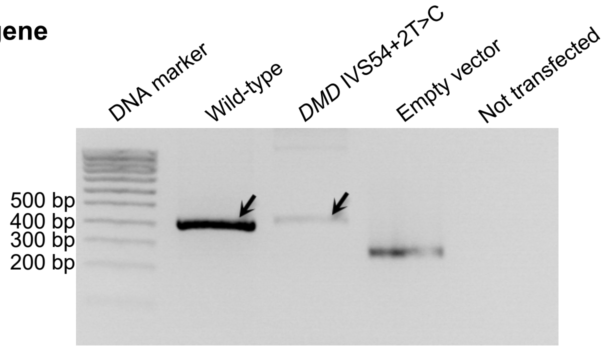

pSPL3 minigene

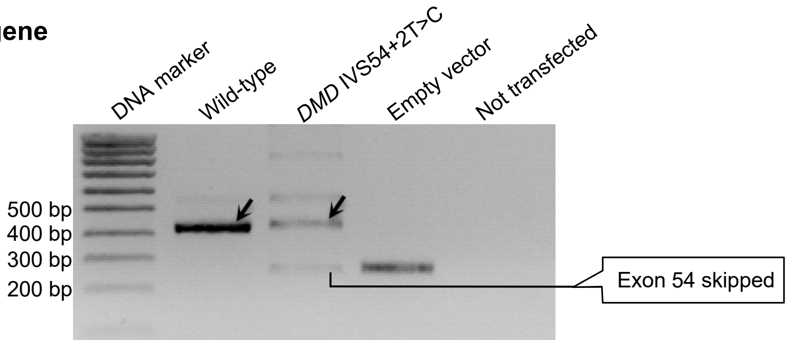

Supplementary Figure S4. Reverse transcription-polymerase chain reaction results from the pET01 and pSPL3 minigene assays with respect to the *DMD* IVS54+2T>C variant. In all panels, wild-type transcripts are indicated by oblique downward pointing arrows.

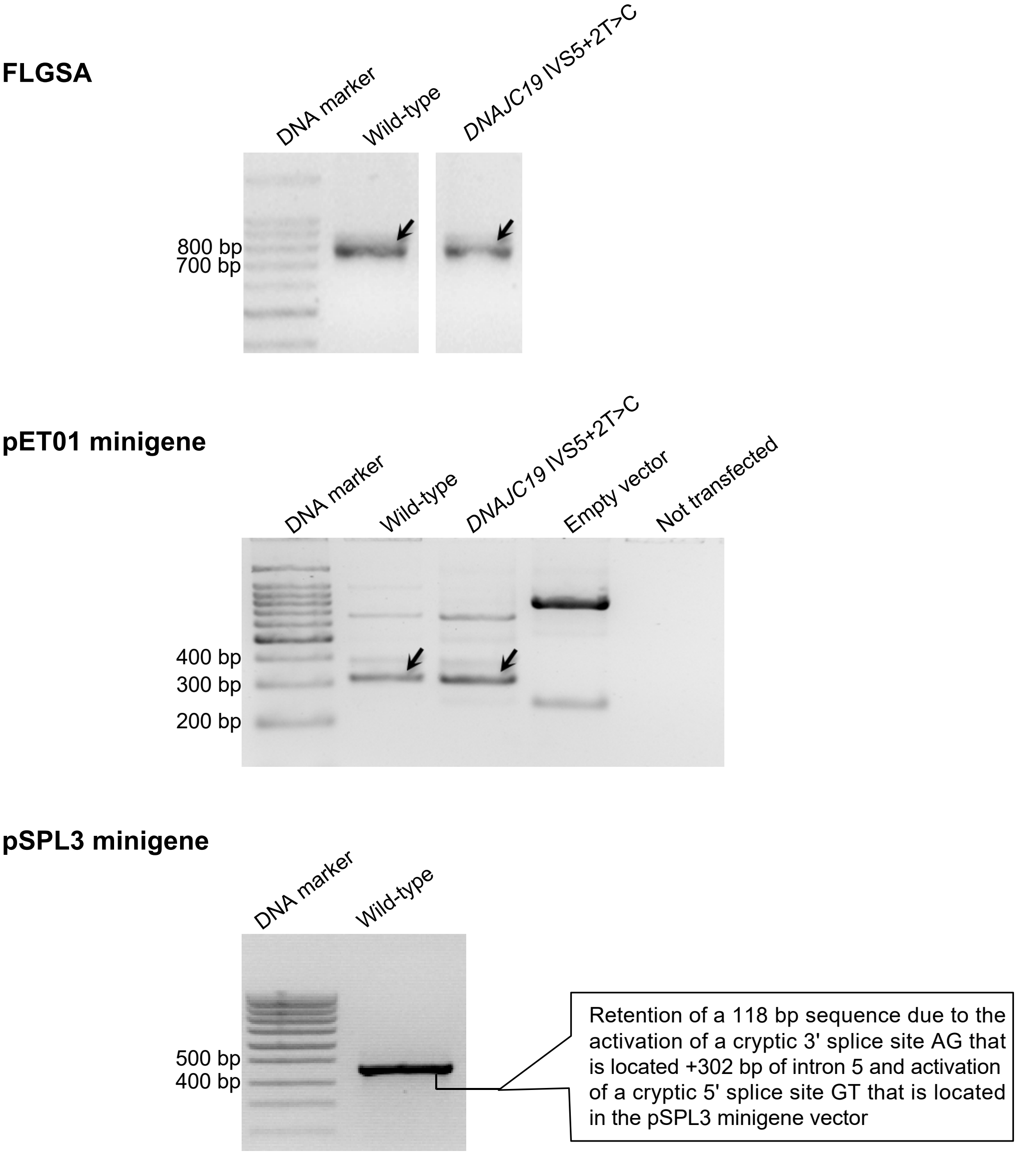

Supplementary Figure S5. Reverse transcription-polymerase chain reaction results from the pET01 and pSPL3 minigene assays with respect to the *DNAJC19* IVS5+2T>C variant. Results from the previously performed full-length gene splicing assay (FLGSA) [12] are included for comparison. In all panels, wild-type transcripts are indicated by oblique downward pointing arrows. The FLGSA data were adapted from Lin et al. (2019) [12] with permission (Copyright 2020 Wiley Periodicals LLC).

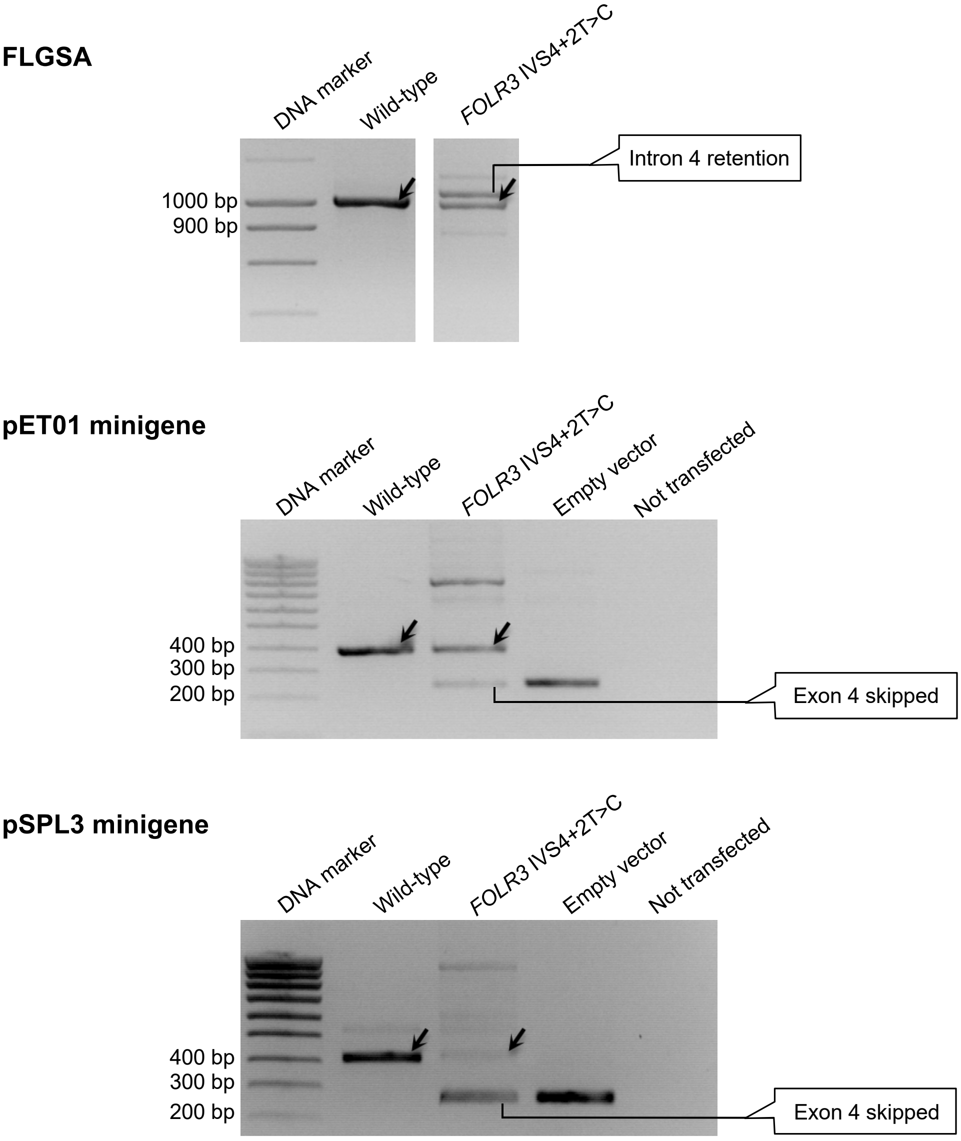

Supplementary Figure S6. Reverse transcription-polymerase chain reaction results from the pET01 and pSPL3 minigene assays with respect to the *FOLR3* IVS4+2T>C variant. Results from the previously performed full-length gene splicing assay (FLGSA) [12] are included for comparison (NB. The aberrant transcript was newly sequenced in this study). In all panels, wild-type transcripts are indicated by oblique downward pointing arrows. The FLGSA data were adapted from Lin et al. (2019) [12] with permission (Copyright 2020 Wiley Periodicals LLC).

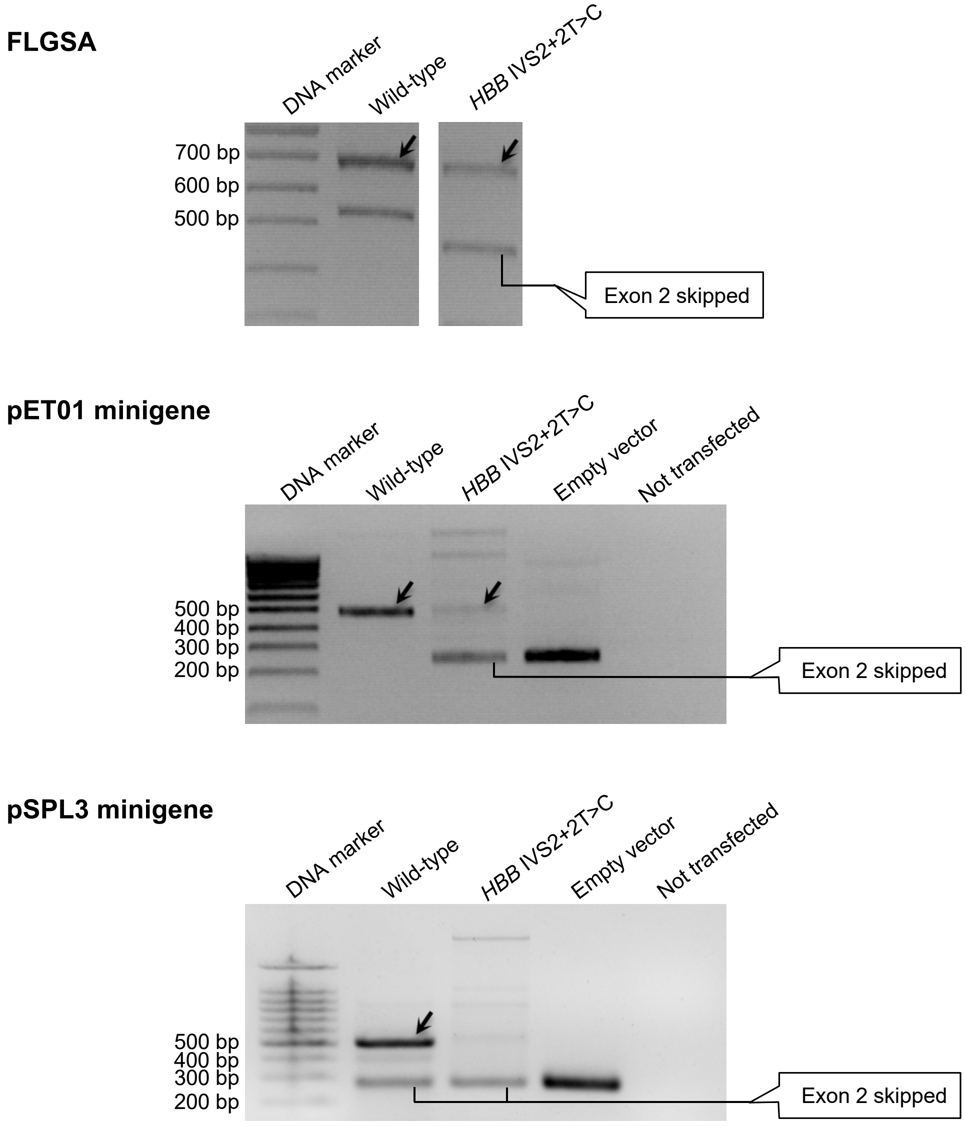

Supplementary Figure S7. Reverse transcription-polymerase chain reaction results from the pET01 and pSPL3 minigene assays with respect to the *HBB* IVS2+2T>C variant. Results from the previously performed full-length gene splicing assay (FLGSA) [12] are included for comparison (NB. The aberrant transcript was newly sequenced in this study). In all panels, wild-type transcripts are indicated by oblique downward pointing arrows. The FLGSA data were adapted from Lin et al. (2019) [12] with permission (Copyright 2020 Wiley Periodicals LLC).

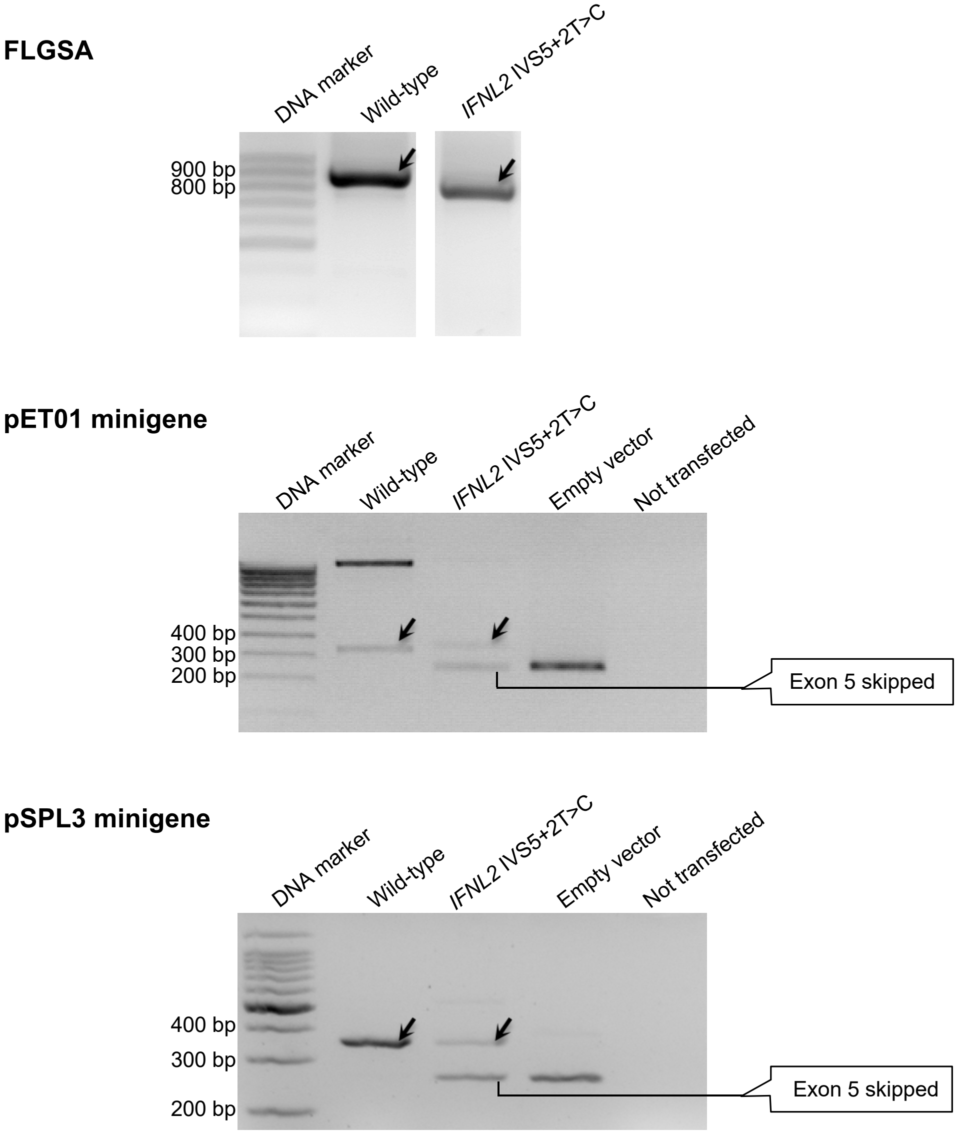

Supplementary Figure S8. Reverse transcription-polymerase chain reaction results from the currently performed pET01 and pSPL3 minigene assays with respect to the *IFNL2* IVS5+2T>C variant. Results from the previously performed full-length gene splicing assay (FLGSA) [12] are included for comparison. In all panels, wild-type transcripts are indicated by oblique downward pointing arrows. The FLGSA data were adapted from Lin et al. (2019) [12] with permission (Copyright 2020 Wiley Periodicals LLC).

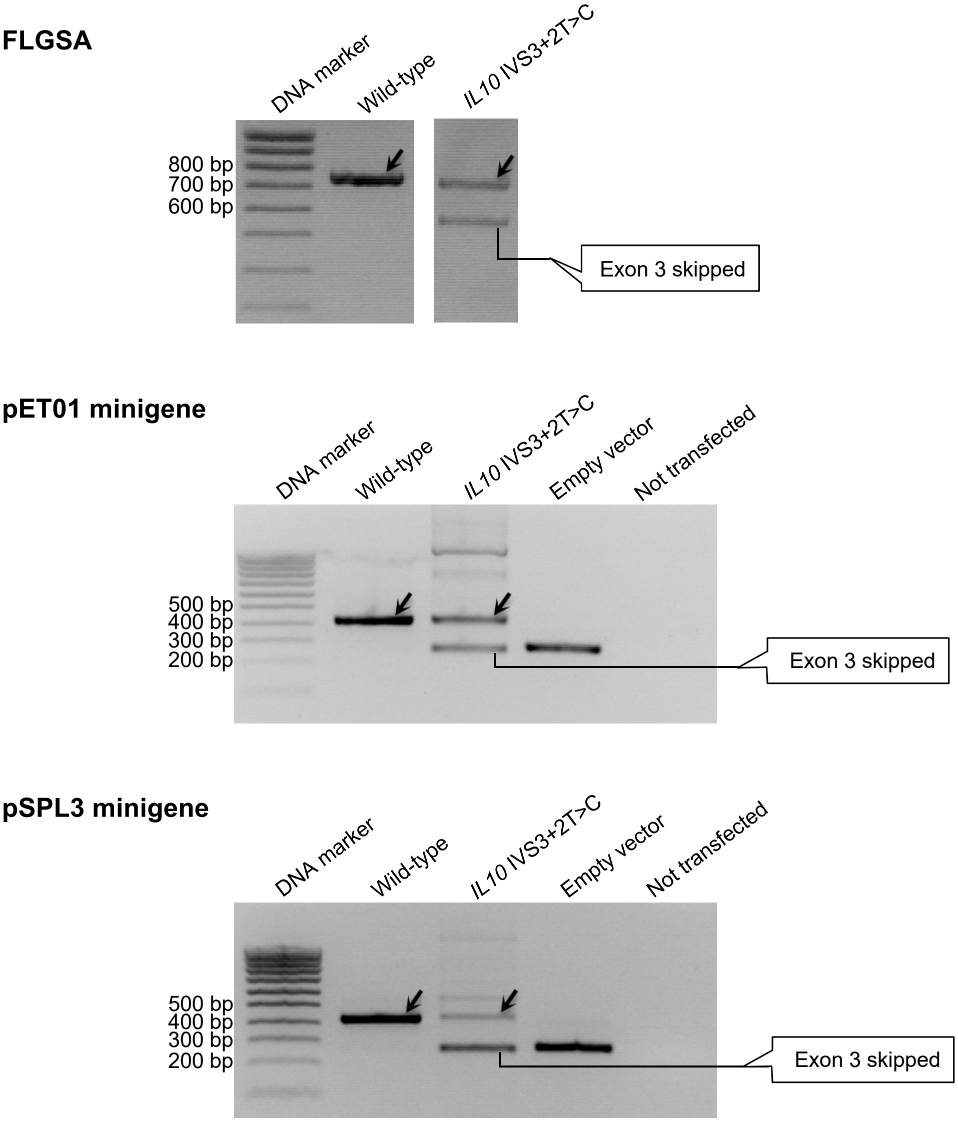

Supplementary Figure S9. Reverse transcription-polymerase chain reaction results from the pET01 and pSPL3 minigene assays with respect to the *IL10* IVS3+2T>C variant. Results from the previously performed full-length gene splicing assay (FLGSA) [12] are included for comparison (NB. The aberrant transcript was newly sequenced in this study). In all panels, wild-type transcripts are indicated by oblique downward pointing arrows. The FLGSA data were adapted from Lin et al. (2019) [12] with permission (Copyright 2020 Wiley Periodicals LLC).

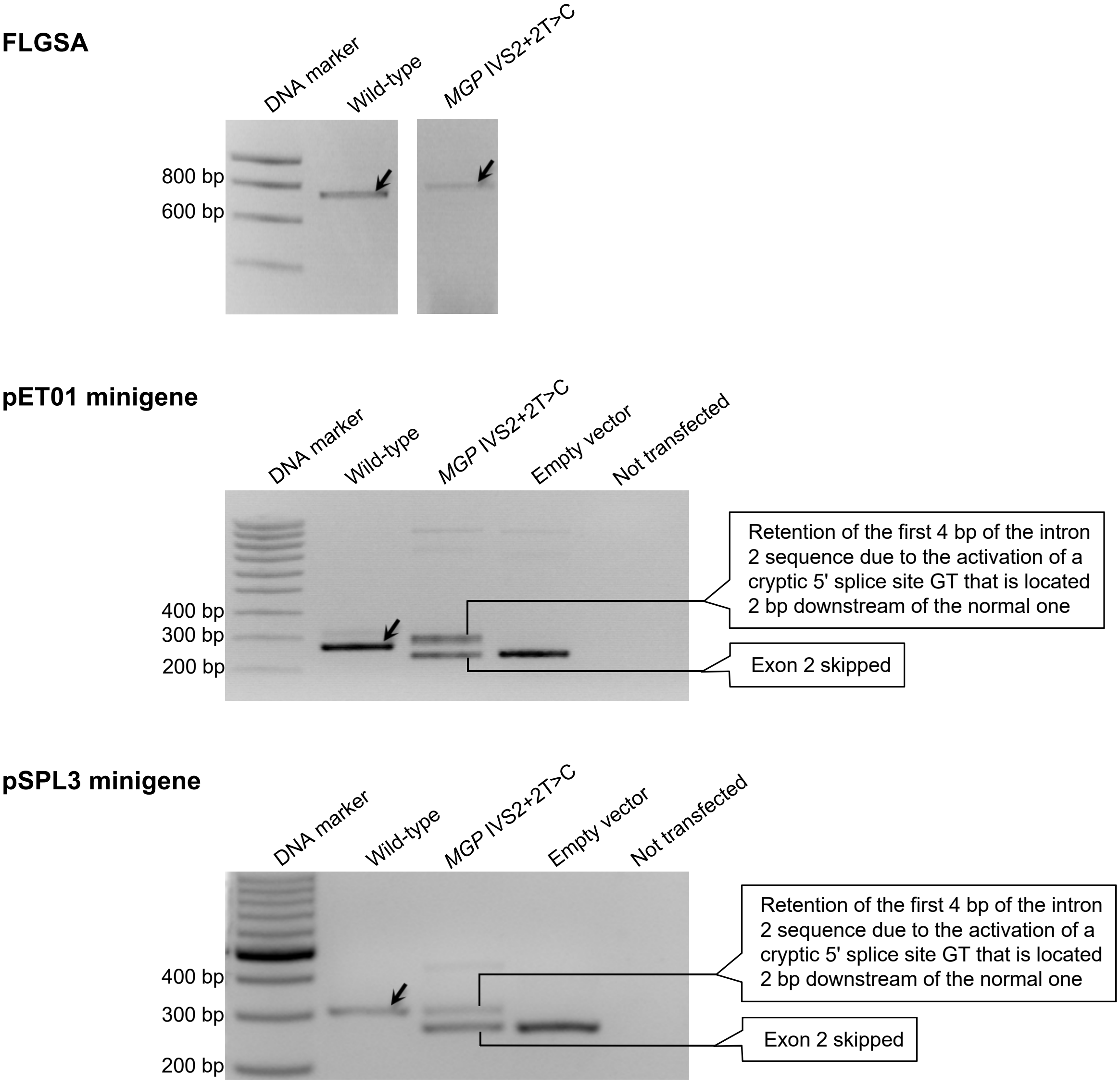

Supplementary Figure S10. Reverse transcription-polymerase chain reaction results from the pET01 and pSPL3 minigene assays with respect to the *MGP* IVS2+2T>C variant. Results from the previously performed full-length gene splicing assay (FLGSA) [12] are included for comparison. In all panels, wild-type transcripts are indicated by oblique downward pointing arrows. The FLGSA data were adapted from Lin et al. (2019) [12] with permission (Copyright 2020 Wiley Periodicals LLC).

pET01 minigene

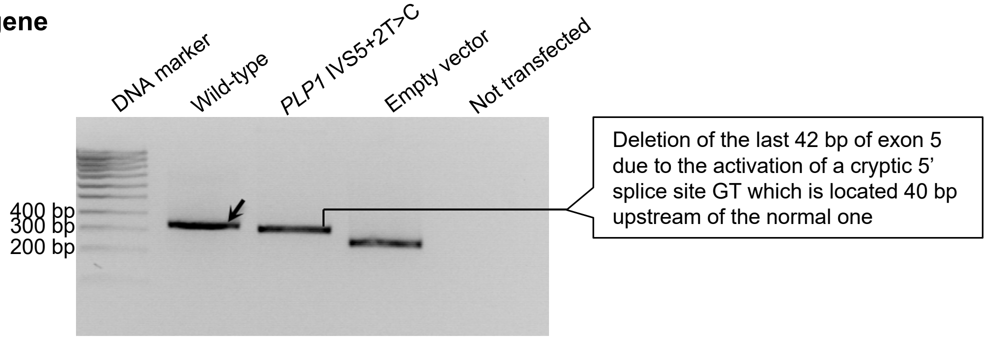

pSPL3 minigene

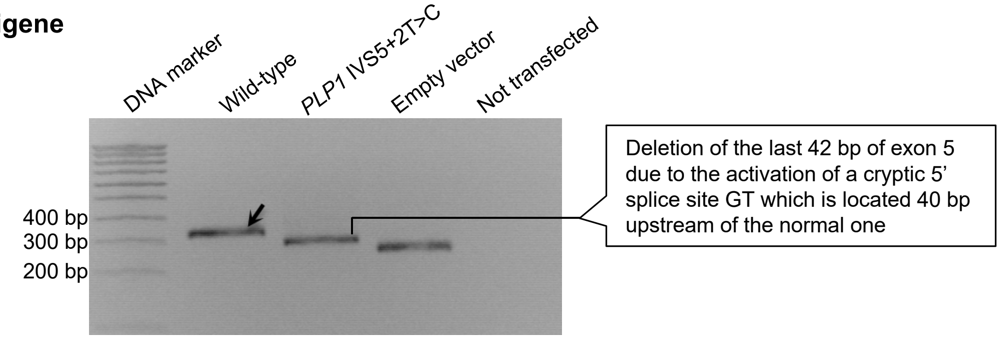

Supplementary Figure S11. Reverse transcription-polymerase chain reaction results from the pET01 and pSPL3 minigene assays with respect to the *PLP1* IVS5+2T>C variant. In all panels, wild-type transcripts are indicated by oblique downward pointing arrows.

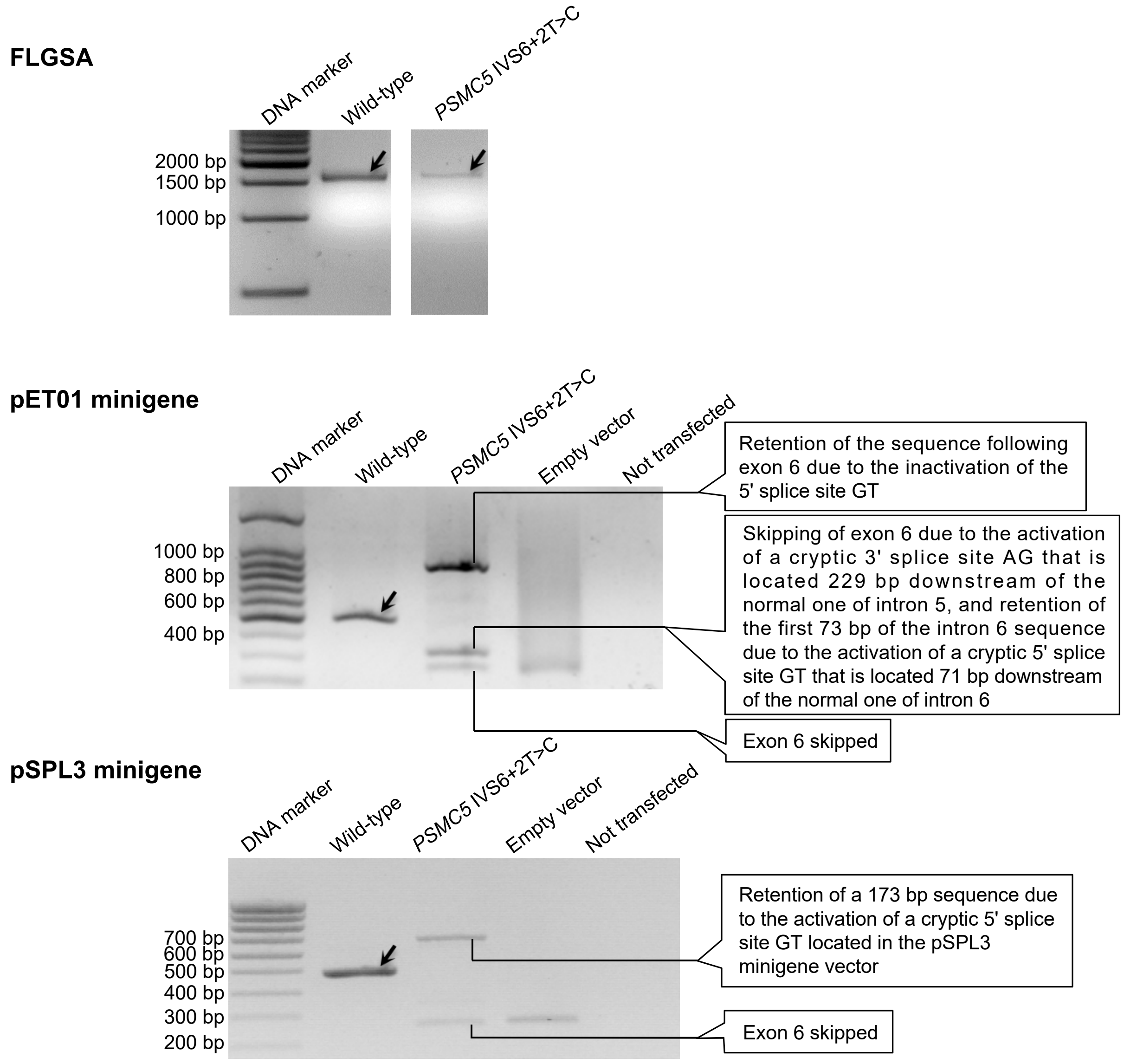

Supplementary Figure S12. Reverse transcription-polymerase chain reaction results from the pET01 and pSPL3 minigene assays with respect to the *PSMC5* IVS6+2T>C variant. Results from the previously performed full-length gene splicing assay (FLGSA) [12] are included for comparison. In all panels, wild-type transcripts are indicated by oblique downward pointing arrows. The FLGSA data were adapted from Lin et al. (2019) [12] with permission (Copyright 2020 Wiley Periodicals LLC).

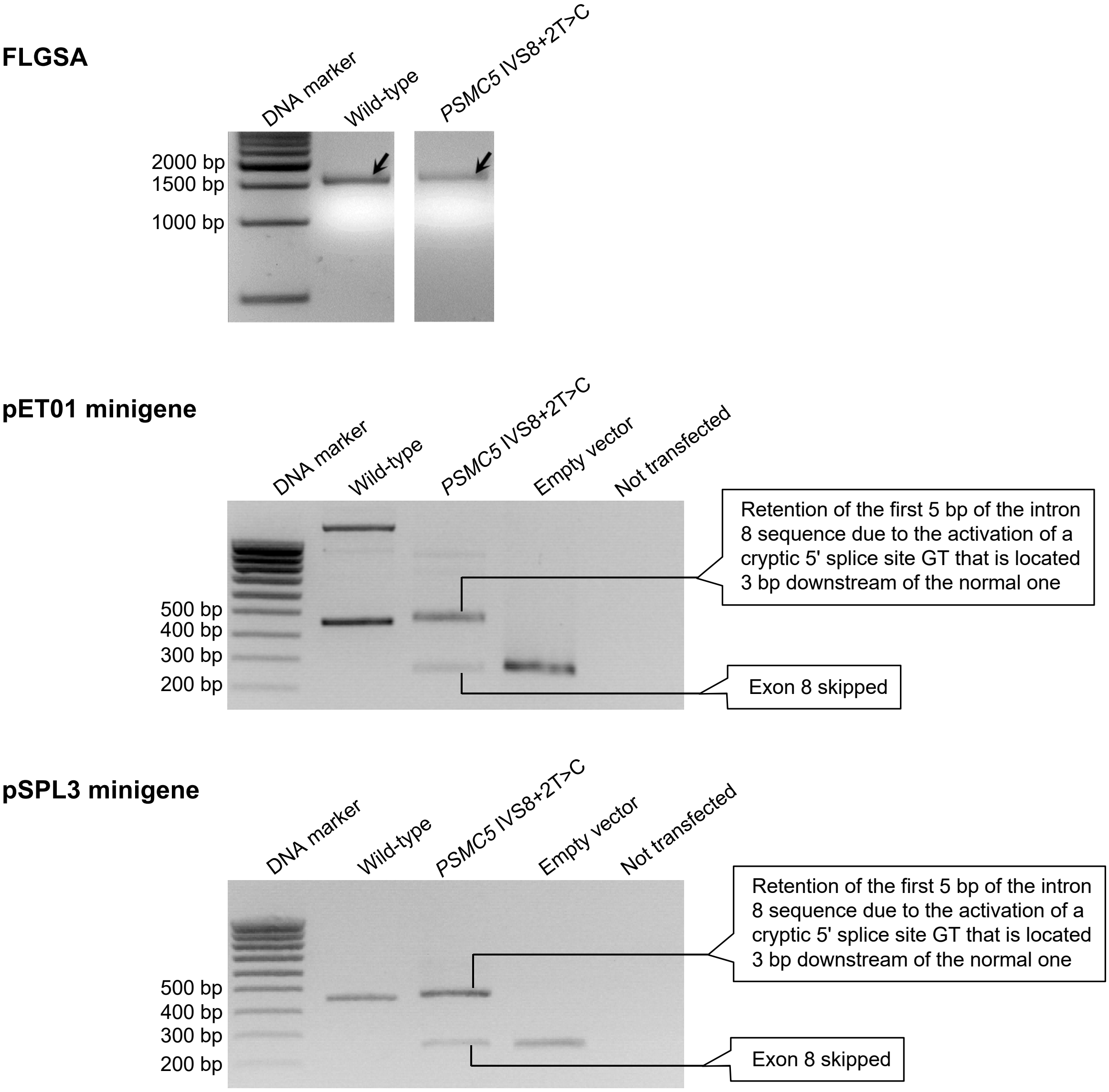

Supplementary Figure S13. Reverse transcription-polymerase chain reaction results from the pET01 and pSPL3 minigene assays with respect to the *PSMC5* IVS8+2T>C variant. Results from the previously performed full-length gene splicing assay (FLGSA) [12] are included for comparison. In all panels, wild-type transcripts are indicated by oblique downward pointing arrows. The FLGSA data were adapted from Lin et al. (2019) [12] with permission (Copyright 2020 Wiley Periodicals LLC).

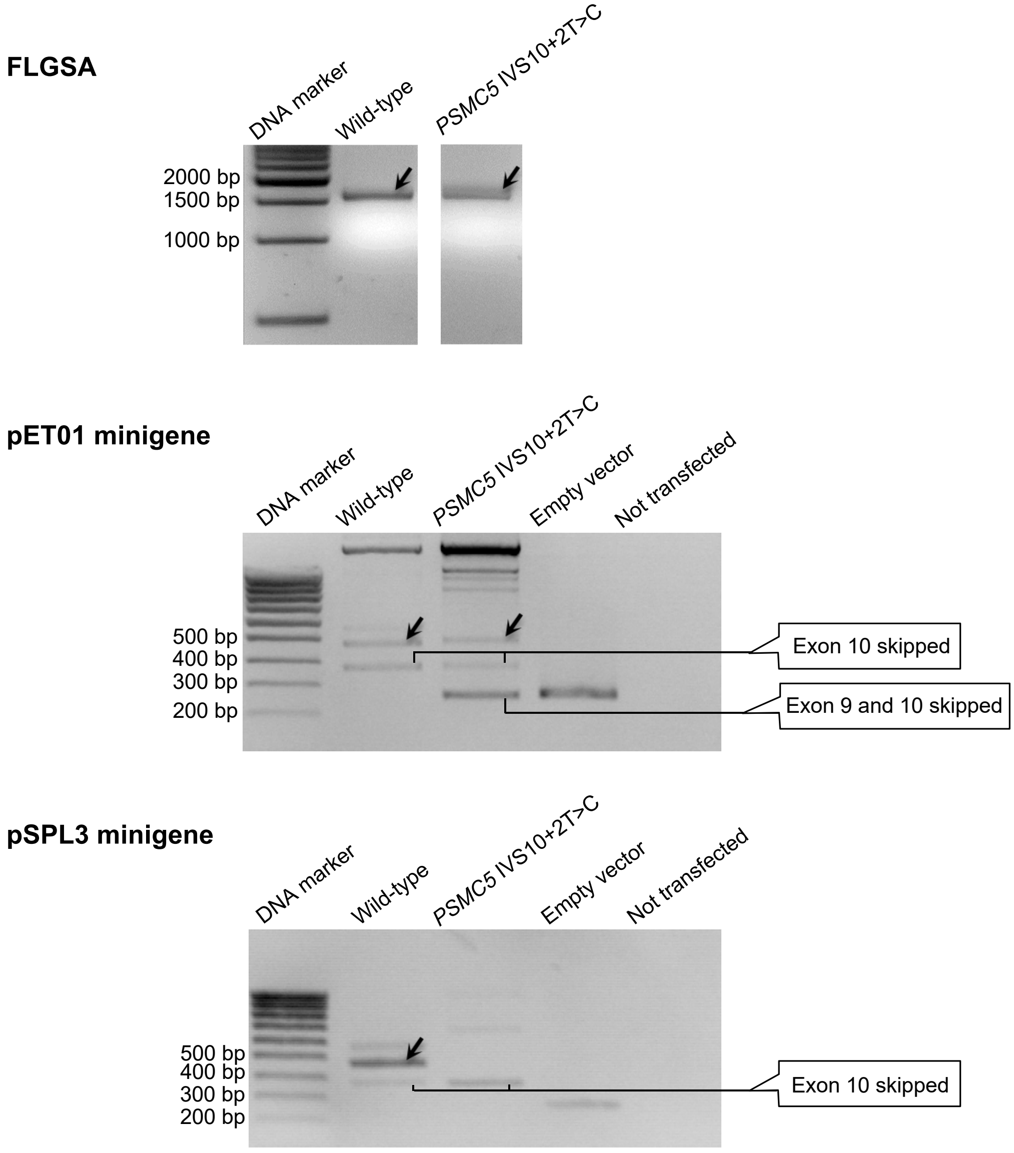

Supplementary Figure S14. Reverse transcription-polymerase chain reaction results from the currently performed pET01 and pSPL3 minigene assays with respect to the *PSMC5* IVS10+2T>C variant. Results from the previously performed full-length gene splicing assay (FLGSA) [12] are included for comparison. In all panels, wild-type transcripts are indicated by oblique downward pointing arrows. The FLGSA data were adapted from Lin et al. (2019) [12] with permission (Copyright 2020 Wiley Periodicals LLC).

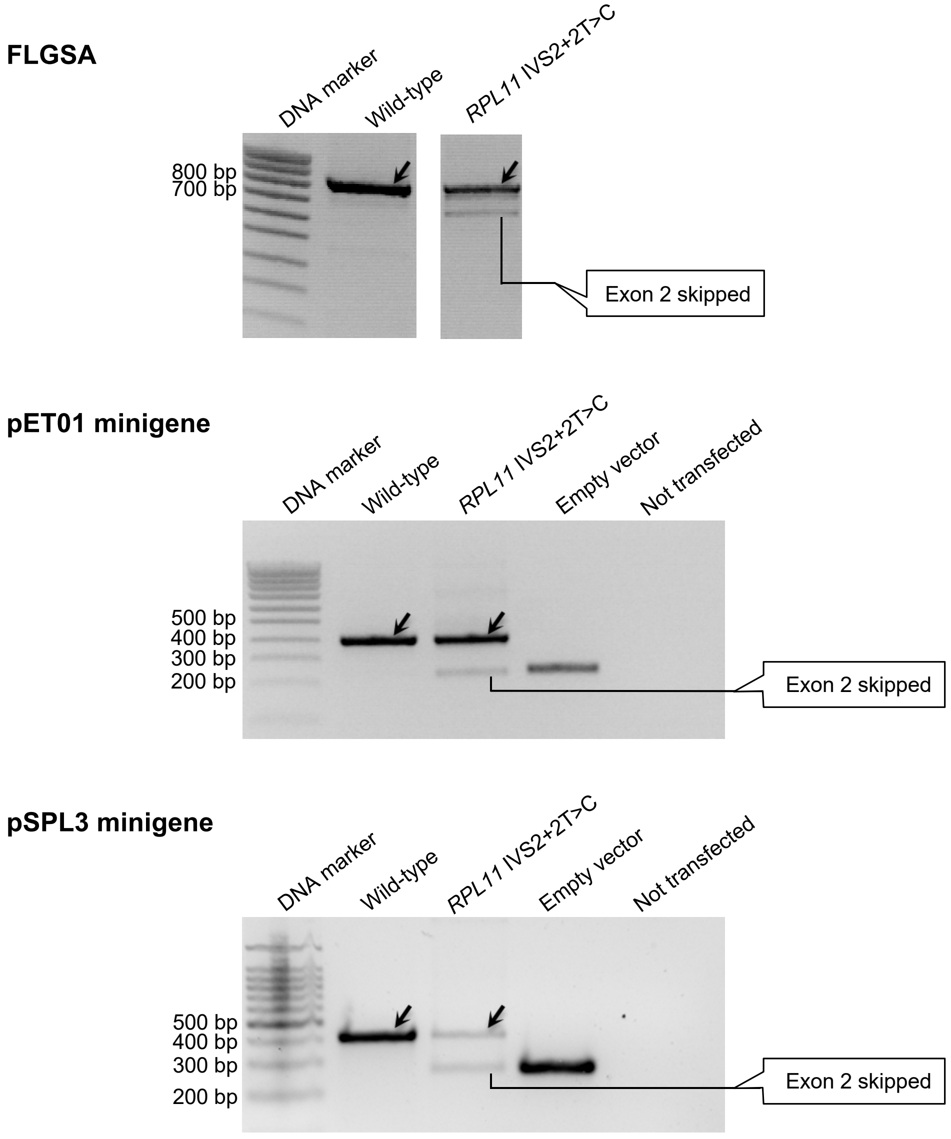

Supplementary Figure S15. Reverse transcription-polymerase chain reaction results from the currently performed pET01 and pSPL3 minigene assays with respect to the *RPL11* IVS2+2T>C variant. Results from the previously performed full-length gene splicing assay (FLGSA) [12] are included for comparison (NB. the aberrant transcript was newly sequenced in this study). In all panels, wild-type transcripts are indicated by oblique downward pointing arrows. The FLGSA data were adapted from Lin et al. (2019) [12] with permission (Copyright 2020 Wiley Periodicals LLC).

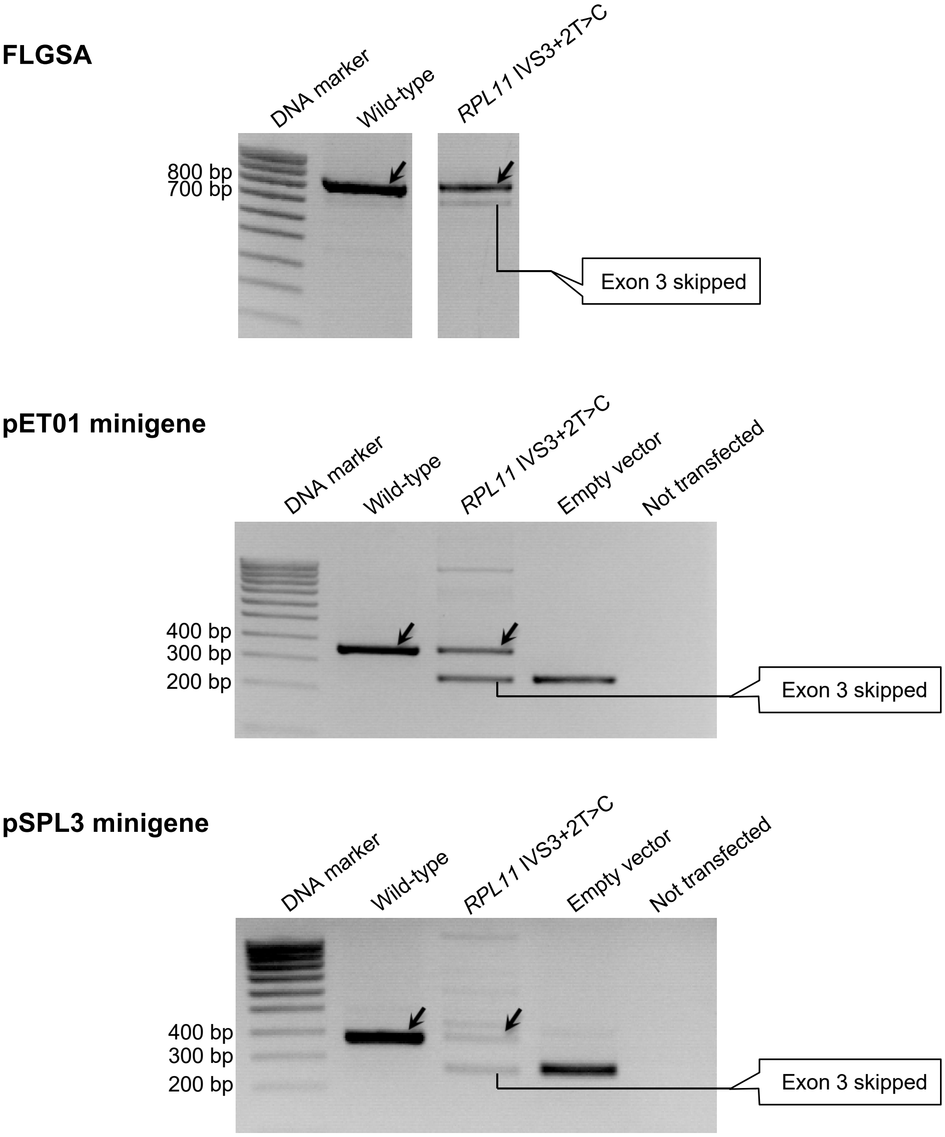

Supplementary Figure S16. Reverse transcription-polymerase chain reaction results from the pET01 and pSPL3 minigene assays with respect to the *RPL11* IVS3+2T>C variant. Results from the previously performed full-length gene splicing assay (FLGSA) [12] are included for comparison (NB. The aberrant transcript was newly sequenced in this study). In all panels, wild-type transcripts are indicated by oblique downward pointing arrows. The FLGSA data were adapted from Lin et al. (2019) [12] with permission (Copyright 2020 Wiley Periodicals LLC).

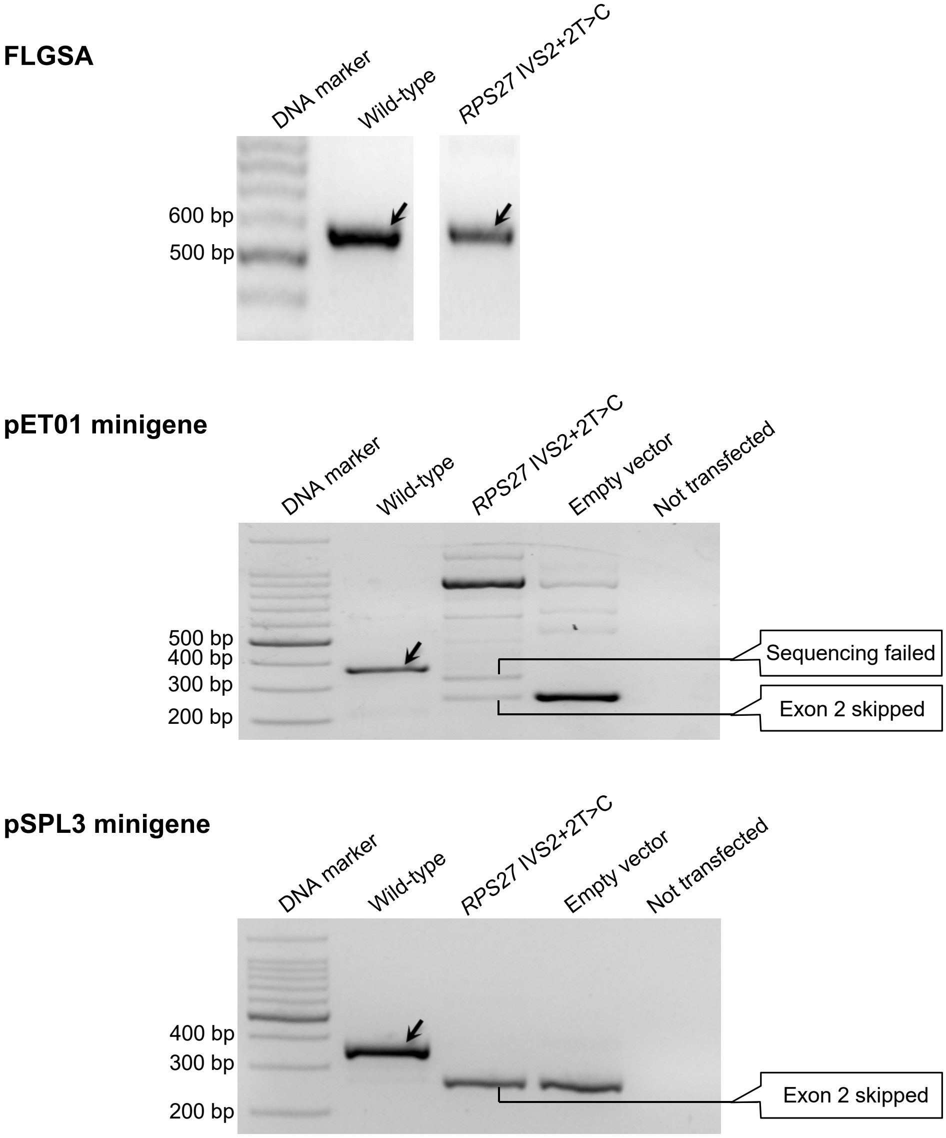

Supplementary Figure S17. Reverse transcription-polymerase chain reaction results from the currently performed pET01 and pSPL3 minigene assays with respect to the *RPS27* IVS2+2T>C variant. Results from the previously performed full-length gene splicing assay (FLGSA) [12] are included for comparison. In all panels, wild-type transcripts are indicated by oblique downward pointing arrows. The FLGSA data were adapted from Lin et al. (2019) [12] with permission (Copyright 2020 Wiley Periodicals LLC).

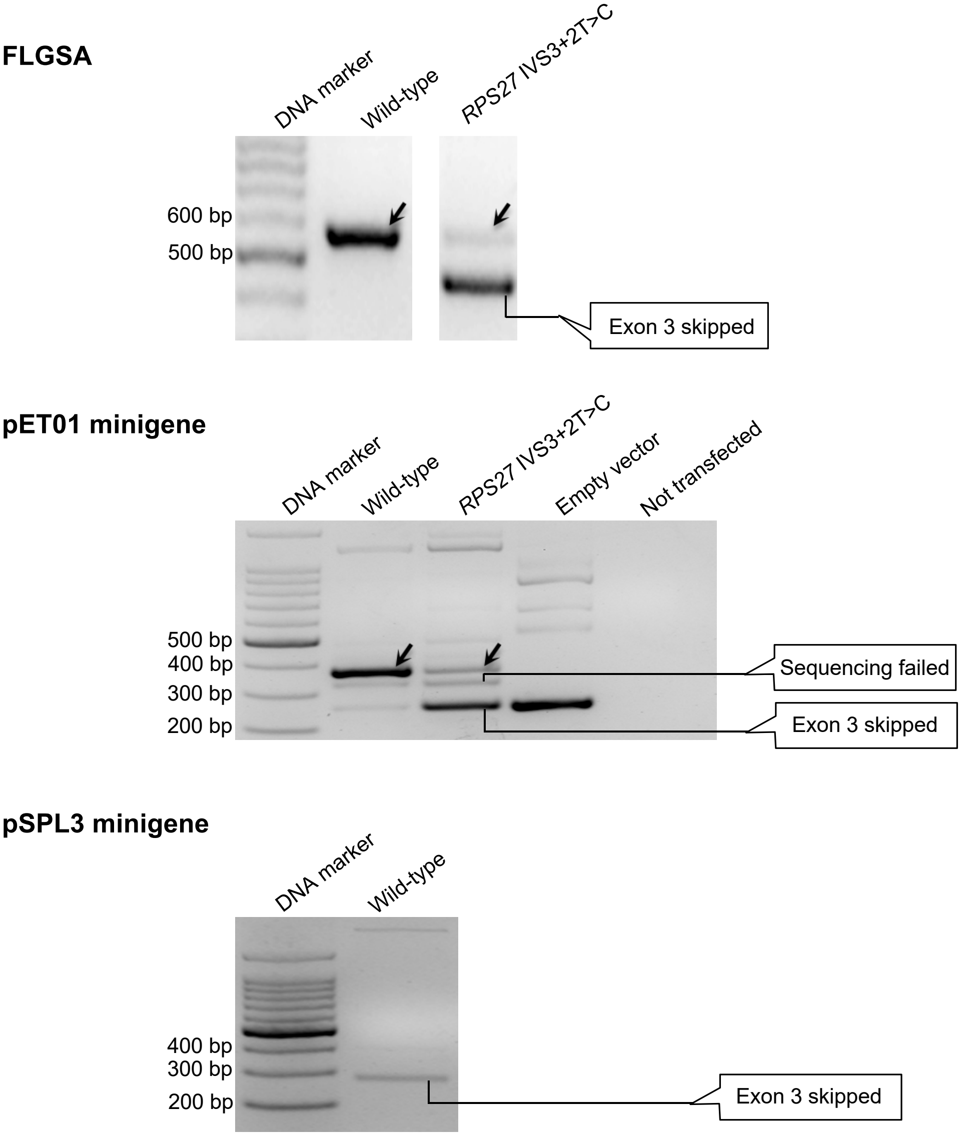

Supplementary Figure S18. Reverse transcription-polymerase chain reaction results from the pET01 and pSPL3 minigene assays with respect to the *RPS27* IVS3+2T>C variant. Results from the previously performed full-length gene splicing assay (FLGSA) [12] are included for comparison (NB. The aberrant transcript was newly sequenced in this study). In all panels, wild-type transcripts are indicated by left oblique downward pointing arrows. The FLGSA data were adapted from Lin et al. (2019) [12] with permission (Copyright 2020 Wiley Periodicals LLC).

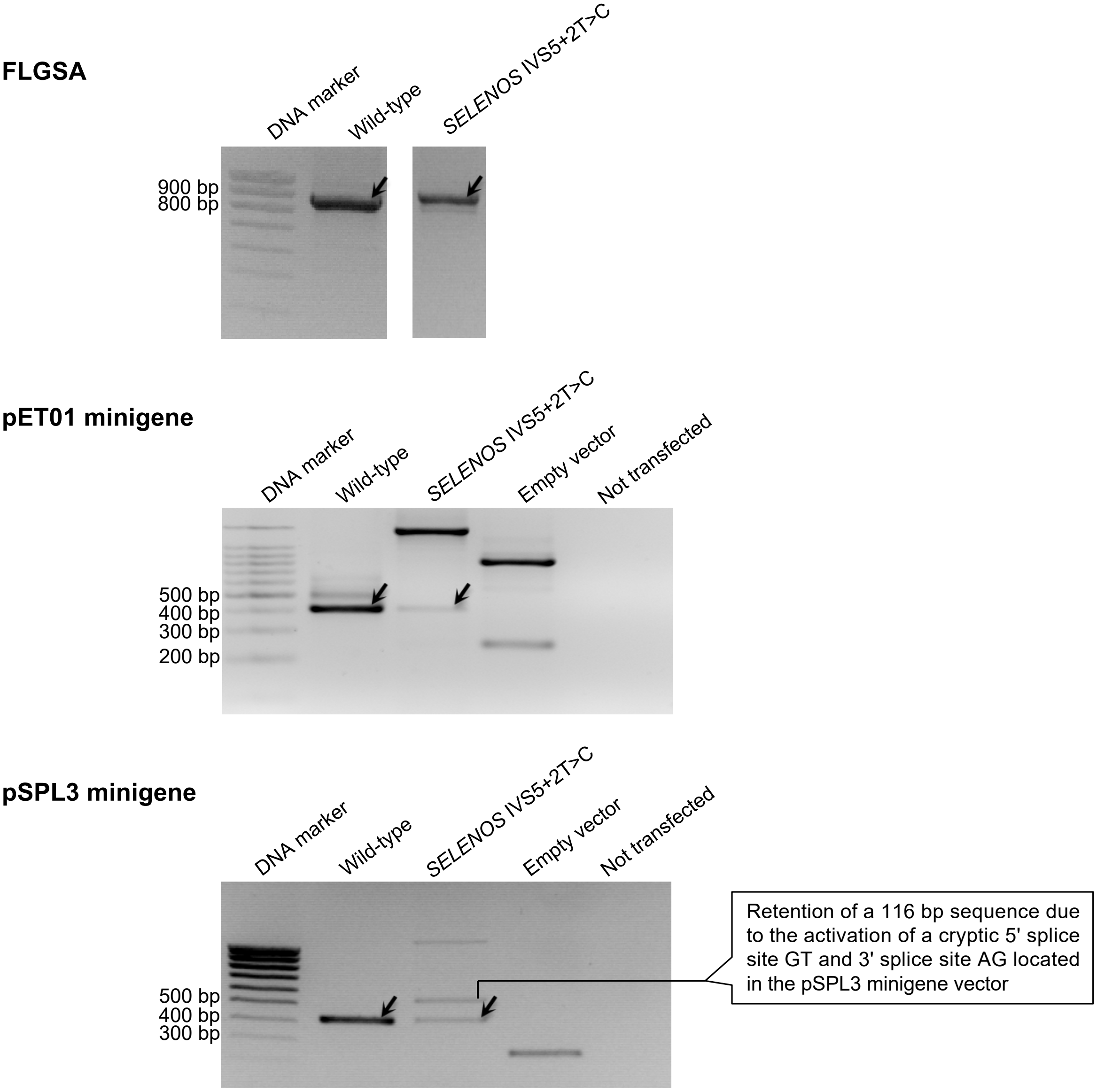

Supplementary Figure S19. Reverse transcription-polymerase chain reaction results from the pET01 and pSPL3 minigene assays with respect to the *SELENOS* IVS5+2T>C variant. Results from the previously performed full-length gene splicing assay (FLGSA) [12] are included for comparison. In all panels, wild-type transcripts are indicated by oblique downward pointing arrows. The FLGSA data were adapted from Lin et al. (2019) [12] with permission (Copyright 2020 Wiley Periodicals LLC).

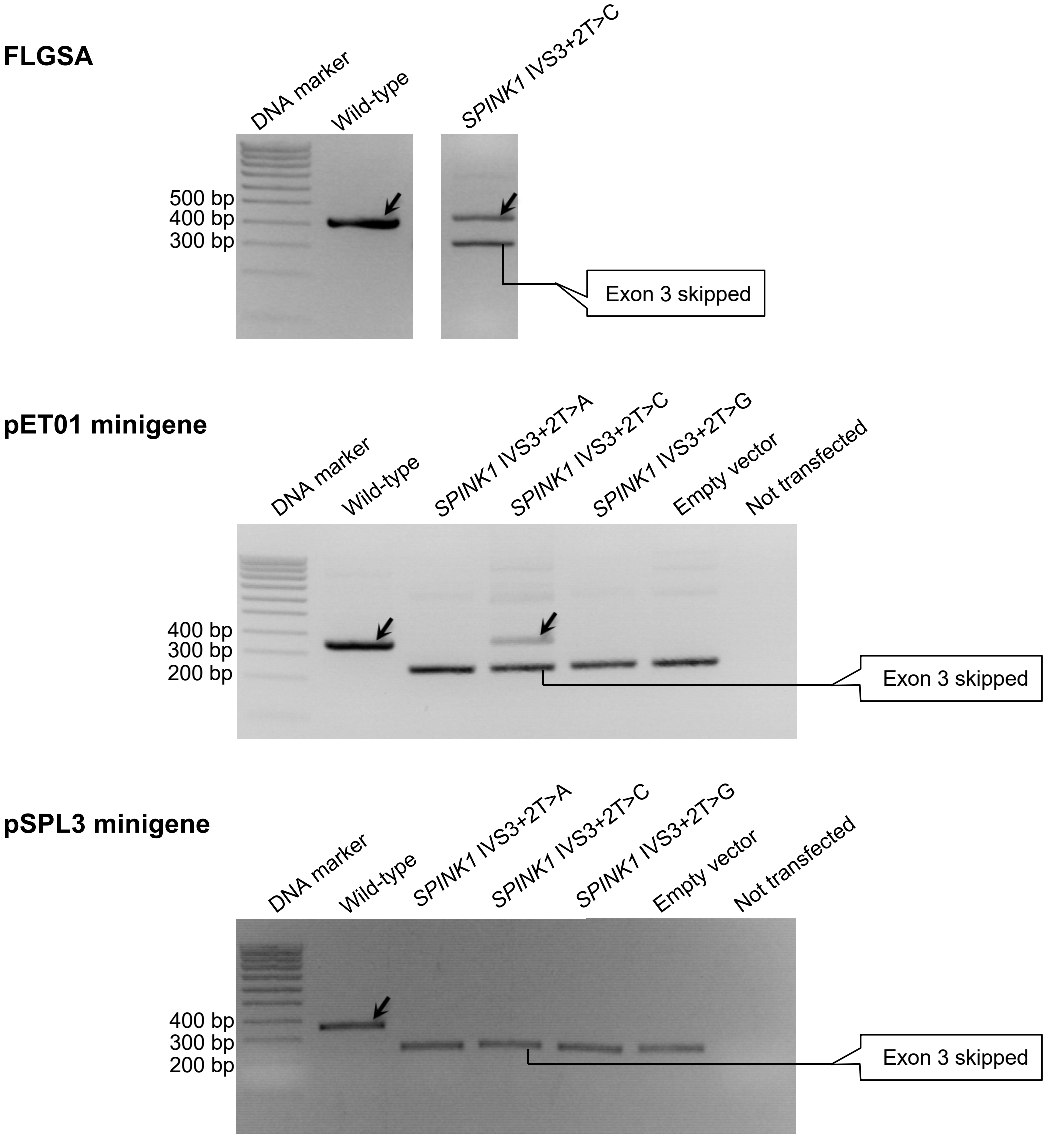

Supplementary Figure S20. Reverse transcription-polymerase chain reaction results from the pET01 and pSPL3 minigene assays with respect to the *SPINK1* IVS3+2T>C variant. Results from the previously performed full-length gene splicing assay (FLGSA) [12] are included for comparison. In all panels, wild-type transcripts are indicated by oblique downward pointing arrows. The FLGSA data were adapted from Lin et al. (2019) [12] with permission (Copyright 2020 Wiley Periodicals LLC). Some other variants not related to this study were also included in the two minigene gels.
